# Treatment with siRNAs is commonly associated with GPX4 up-regulation and target knockdown-independent sensitization to ferroptosis

**DOI:** 10.1126/sciadv.adk7329

**Published:** 2024-03-15

**Authors:** Anne von Mässenhausen, Marlena Nastassja Schlecht, Kristina Beer, Francesca Maremonti, Wulf Tonnus, Alexia Belavgeni, Shubhangi Gavali, Karolin Flade, Joel S. Riley, Nadia Zamora Gonzalez, Anne Brucker, Jorunn Naila Becker, Mirela Tmava, Claudia Meyer, Mirko Peitzsch, Christian Hugo, Florian Gembardt, Jose Pedro Friedmann Angeli, Stefan R. Bornstein, Stephen W. G. Tait, Andreas Linkermann

**Affiliations:** ^1^ Division of Nephrology, Department of Internal Medicine III, University Hospital Carl Gustav Carus at the Technische Universität Dresden, 01307 Dresden, Germany.; ^2^ Institute of Clinical Chemistry and Clinical Pharmacology, University Hospital Bonn, 53127 Bonn, Germany.; ^3^ Cancer Research UK Beatson Institute, Switchback Road, Glasgow G61 1BD, UK.; ^4^ School of Cancer Sciences, University of Glasgow, Switchback Road, Glasgow G61 1BD, UK.; ^5^ Biocenter Innsbruck (CCB), Medical University Innsbruck, Division of Developmental Immunology, Innrain 80, 6020 Innsbruck, Austria.; ^6^ Institute of Clinical Chemistry and Laboratory Medicine, University Hospital Carl Gustav Carus at the Technische Universität Dresden, 01307 Dresden, Germany.; ^7^ Rudolf Virchow Center for Integrative and Translational Bioimaging, Chair of Translational Cell Biology, University of Würzburg, 97080 Würzburg, Germany.; ^8^ Department of Internal Medicine 3, University Hospital Carl Gustav Carus at the Technische Universität Dresden, Dresden, Germany.; ^9^ Diabetes and Nutritional Sciences, King's College London, London, UK.; ^10^ Center for Regenerative Therapies, Technische Universität Dresden, Dresden, Germany.; ^11^ Paul Langerhans Institute Dresden of Helmholtz Centre Munich at University Clinic Carl Gustav Carus of TU Dresden Faculty of Medicine, Dresden, Germany.; ^12^ Lee Kong Chian School of Medicine, Nanyang Technological University, Singapore, Singapore.; ^13^ Division of Nephrology, Department of Medicine, Albert Einstein College of Medicine, Bronx, NY, USA.

## Abstract

Small interfering RNAs (siRNAs) are widely used in biomedical research and in clinical trials. Here, we demonstrate that siRNA treatment is commonly associated with significant sensitization to ferroptosis, independently of the target protein knockdown. Genetically targeting mitochondrial antiviral-signaling protein (MAVS) reversed the siRNA-mediated sensitizing effect, but no activation of canonical MAVS signaling, which involves phosphorylation of IkBα and interferon regulatory transcription factor 3 (IRF3), was observed. In contrast, MAVS mediated a noncanonical signal resulting in a prominent increase in mitochondrial ROS levels, and increase in the BACH1/pNRF2 transcription factor ratio and GPX4 up-regulation, which was associated with a 50% decrease in intracellular glutathione levels. We conclude that siRNAs commonly sensitize to ferroptosis and may severely compromise the conclusions drawn from silencing approaches in biomedical research. Finally, as ferroptosis contributes to a variety of pathophysiological processes, we cannot exclude side effects in human siRNA-based therapeutical concepts that should be clinically tested.

## Teaser

A commonly overlooked sensitization to ferroptosis upon siRNA transfection, mediated through up-regulation of GPX4, is described.

## INTRODUCTION

Small interfering RNAs (siRNAs) are widely used as tool compounds in biomedical research and recently have entered clinical trials for potential therapeutic benefit (*1*–*4*). However, treatment of a cell with an siRNA markedly alters the metabolic state (*5*, *6*), and detailed mechanisms of the sensitization to cellular death and survival are understudied. Given the wide range of siRNA applications, it is surprising that even in cultured cells, changes to classical pathways of regulated cell death, such as apoptosis, necroptosis, pyroptosis, and ferroptosis, have not been investigated.

Ferroptosis is a subtype of regulated necrosis (*7*) that is catalyzed by free iron and results in lipid peroxidation of polyunsaturated fatty acids in the plasma membrane to cause plasma membrane rupture (*8*, *9*), the state of a cell that defines a necrotic phenotype (*10*). Pathophysiologically, ferroptosis is the driving mechanism in myocardial infarction (*11*, *12*), stroke (*13*, *14*), acute kidney injury (*15*), and other common diseases (*16*).

## RESULTS

**Fig. 1.**
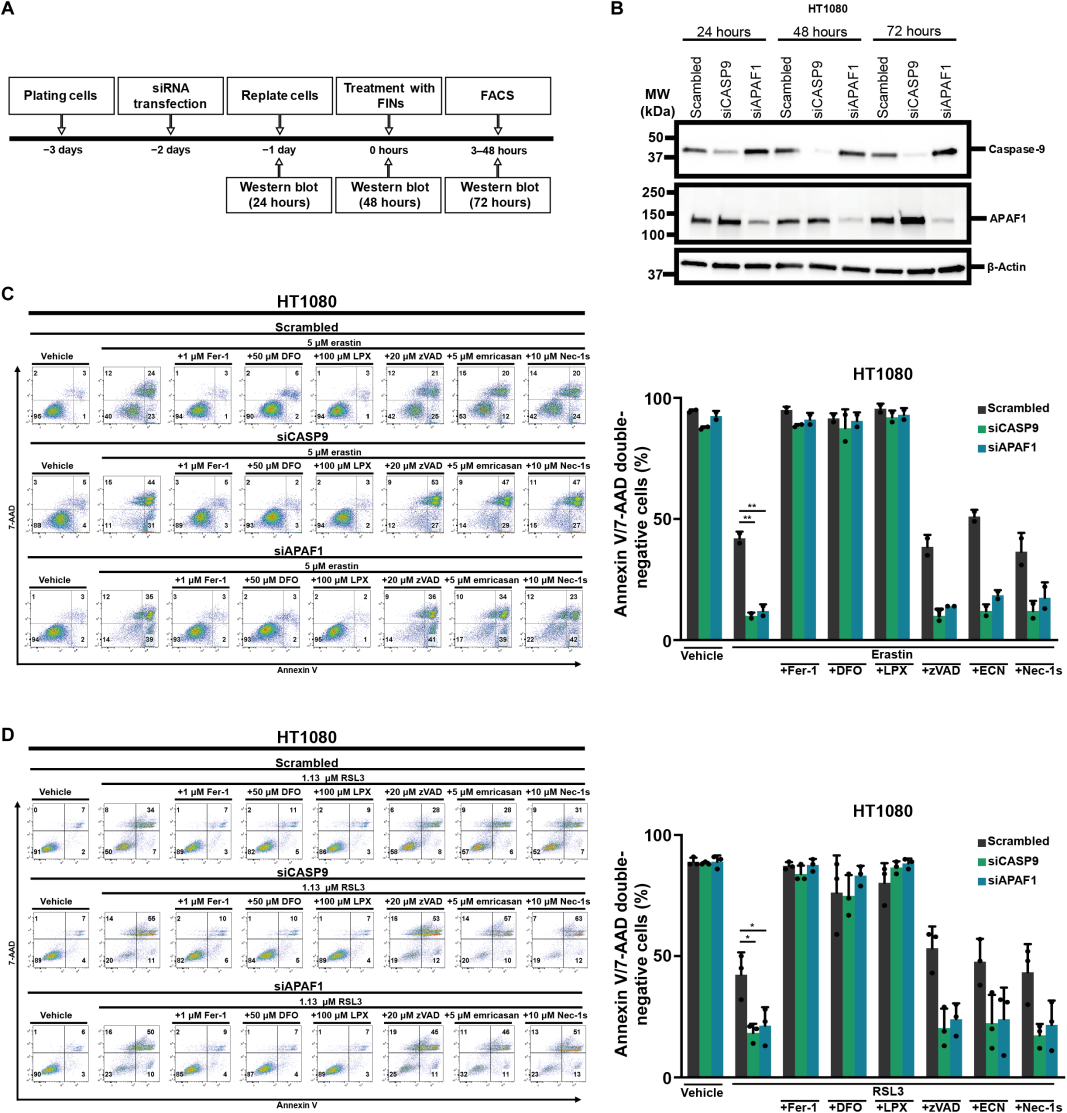
siRNA against caspase-9 and APAF1 sensitize toward ferroptosis. (**A**) Experimental setup for knockdown experiments: HT1080 cells were plated 1 day before siRNA transfection. After 24 hours, cells were replated and ferroptosis was induced another day later (48 hours after siRNA knockdown). 7-AAD and annexin V were read out by fluorescence-activated cell sorting (FACS). (**B**) Western blot analysis to determine knockdown efficacy of caspase-9 and APAF1 24, 48, and 72 hours after siRNA treatment. (**C**) Caspase-9 or APAF1 was knocked down in HT1080 cells before treating them with erastin. 7-AAD and annexin V were read out by FACS. Primary FACS plots and respective quantifications of annexin V/7-AAD double-negative cells are demonstrated. (**D**) Caspase-9 or APAF1 was knocked down in HT1080 cells before treating them with RSL3. 7-AAD and annexin V were read out by FACS. Primary FACS plots and respective quantifications of annexin V/7-AAD double-negative cells are demonstrated. The graphs show means ± SD. Statistical analysis was performed using one-way ANOVA. *P ≤ 0.05, **P ≤ 0.01. DFO, deferoxamine; LPX,: liproxstatin; ECN, emricasan.

Our starting point in the present study was the assumption that caspase-9, recently identified as a hotspot for SNPs in the genomes of chronic kidney disease patients, may be involved in ferroptosis (*17*). Given that ferroptosis drives kidney disease (*18*), we tested the role of caspase-9 in ferroptosis in general. We treated HT1080 cells for 24, 48, and 72 hours with a caspase-9 targeting siRNA and confirmed the knockdown efficacy by Western blot (Fig. 1, A and B). We followed a similar approach with APAF-1, a scaffold protein in the formation of the apoptosome (*19*) downstream of mitochondrial outer membrane permeabilization (MOMP) (*20*, *21*). We then treated HT1080 cells with a type 1 ferroptosis inducer (FIN), erastin, for 24 hours following knockdown. As demonstrated in Fig. 1C, both siRNA treatments sensitized cells to annexin V/7-AAD double positivity in FACS (fluorescence-activated cell sorting), compared with controls. Similar sensitizing effects were detected when ferroptosis was induced by the GPX4 inhibitor RSL3 (Fig. 1D). Given these results, we tested type 3 and type 4 FINs FIN56 and FINO2, respectively. Figure S1 (A and B) demonstrates sensitization to ferroptosis in all cases investigated. We repeated experiments to test sensitization to ferroptosis after siRNA treatment in the renal tubular cell line CD10-135 with similar results regarding the sensitization. This confirmed that siRNA-mediated sensitization to ferroptosis is not a cell line–specific effect (fig. S2). These data suggested that siRNAs against caspase-9 and APAF-1 sensitize HT1080 cells to ferroptosis.

**Fig. 2.**
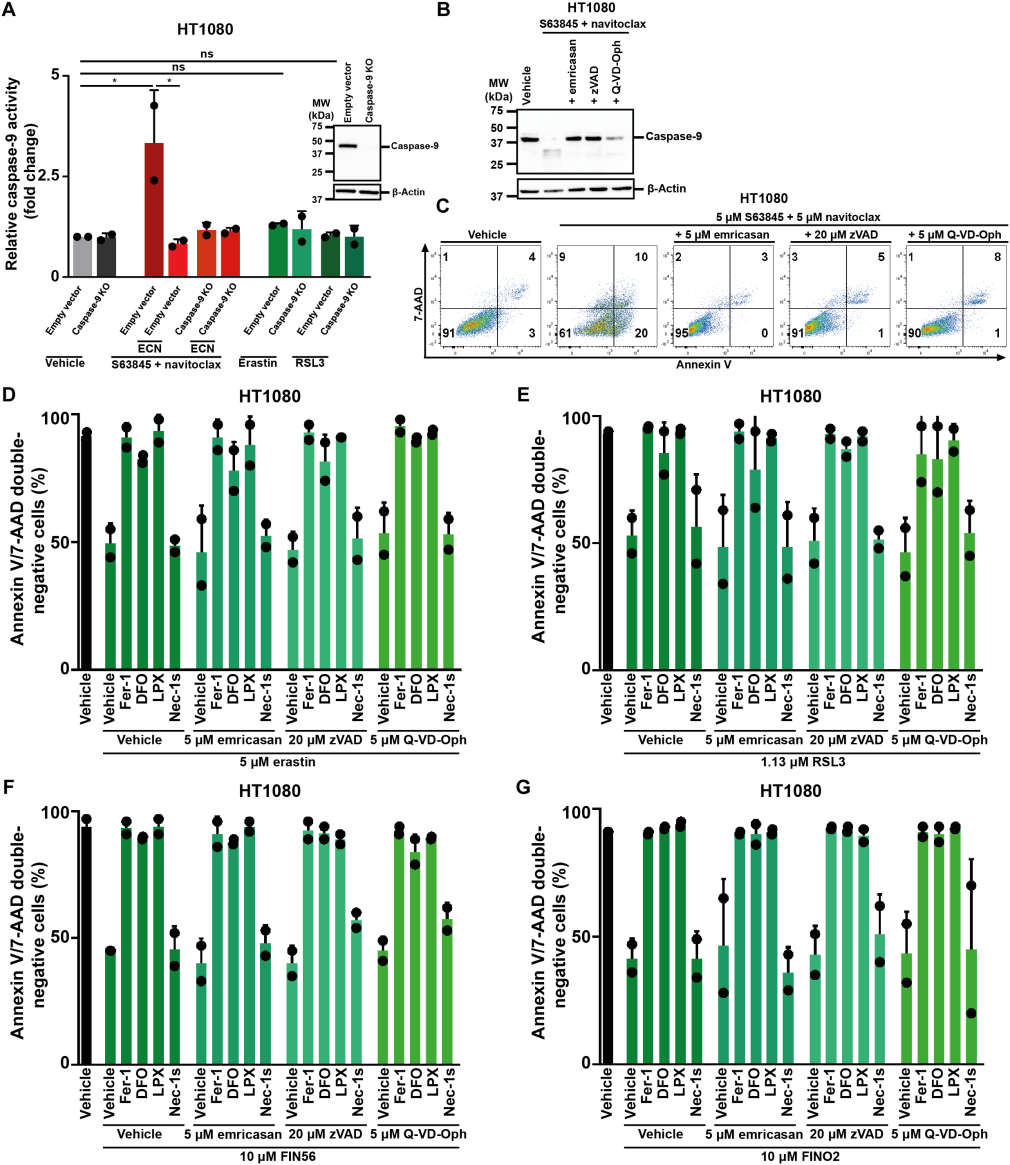
Caspase-9 activity plays no role in sensitization toward ferroptosis. (**A**) In HT1080 wild-type or *caspase-9* knockout cells, either apoptosis (S63845 and Navitoclax) or ferroptosis (erastin or RSL3) was induced before measuring caspase-9 activity. (**B** and **C**) Apoptosis was induced in HT1080 cells using S63845 and Navitoclax with or without cotreatment of different caspase inhibitors. Caspase-9 cleavage was investigated using Western blotting and 7-AAD and annexin V were read out by FACS. (**D**) HT1080 cells were treated with different caspase inhibitors before inducing ferroptosis using erastin. 7-AAD and annexin V were read out by FACS. Quantifications of annexin V/7-AAD double-negative cells are demonstrated. (**E**) HT1080 cells were treated with different caspase inhibitors before inducing ferroptosis using RSL3. 7-AAD and annexin V were read out by FACS. Quantifications of annexin V/7-AAD double-negative cells are demonstrated. (**F**) HT1080 cells were treated with different caspase inhibitors before inducing ferroptosis using FIN56. 7-AAD and annexin V were read out by FACS. Quantifications of annexin V/7-AAD double-negative cells are demonstrated. (**G**) HT1080 cells were treated with different caspase inhibitors before inducing ferroptosis using FINO2. 7-AAD and annexin V were read out by FACS. Quantifications of annexin V/7-AAD double-negative cells are demonstrated. The graphs show means ± SD. Statistical analysis was performed using Student’s *t* test or one-way ANOVA. *P ≤ 0.05; ns, not significant. DFO, deferoxamine; LPX, liproxstatin.

To test the direct role that caspase-9 catalytic activity plays in ferroptosis sensitization, we treated HT1080 cells with a combination of S63845 (an MCL-1 inhibitor) and navitoclax (a combined inhibitor of BCL-xL and BCL-2). As expected, the combined treatment with S63845 and navitoclax increased caspase activity (Fig. 2A), resulted in capsase-9 cleavage (Fig. 2B), and increased annexin V positivity (Fig. 2C), and this effect was entirely reversed by the pan-caspase inhibitors emricasan (Fig. 2, A to C), zVAD-fmk (hereafter referred to as zVAD), and q-VD (Fig. 2, B and C). Caspase-9 activity remained unchanged upon erastin or RSL3 treatment (Fig. 2A). We next tested the effect of the pan-caspase inhibitors emricasan, zVAD, and q-VD alongside the necroptosis-inhibitor Nec-1s and the known ferrostatins, ferrostatin-1 (Fer-1), deferoxamine (DFO), and liproxstatin-1 (LPX), upon induction of ferroptosis by erastin (Fig. 2D), RSL3 (Fig. 2E), FIN56 (Fig. 2F), and FINO2 (Fig. 2G). Collectively, these results exclude the role of catalytic caspase activation as the ferroptosis-sensitizing mechanism.

**Fig. 3.**
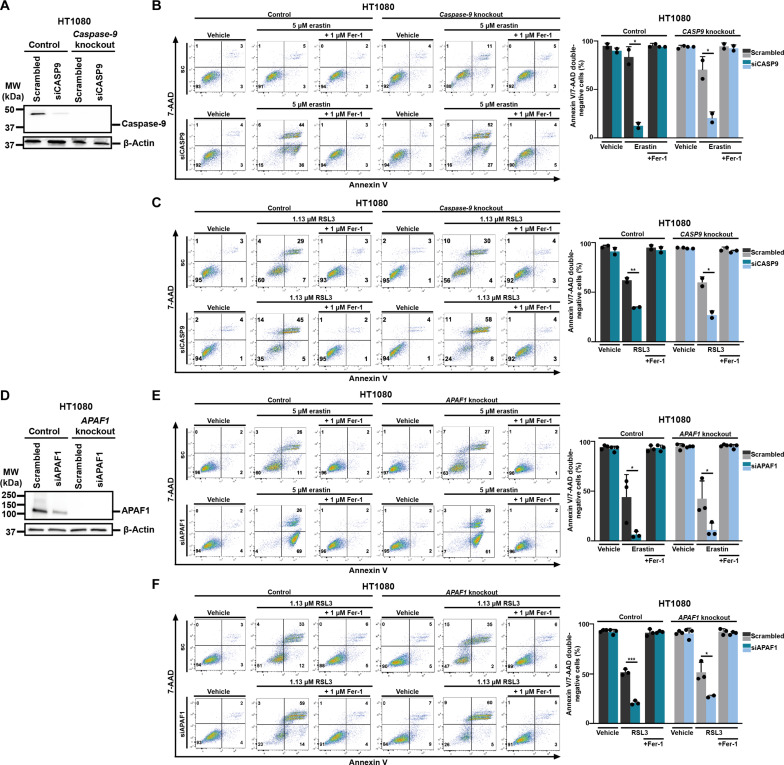
siRNA against caspase-9 and APAF1 sensitizes toward ferroptosis in respective knockout cells. (**A**) Western blot analysis to determine caspase-9 knockdown and knockout efficacy in HT1080 cells. (**B**) Caspase-9 was knocked down in HT1080 control and *caspase-9* knockout cells before treating them with erastin. 7-AAD and annexin V were read out by FACS. Primary FACS plots and respective quantifications of annexin V/7-AAD double-negative cells are demonstrated. (**C**) Caspase-9 was knocked down in HT1080 control and *caspase-9* knockout cells before treating them with RSL3. 7-AAD and annexin V were read out by FACS. Primary FACS plots and respective quantifications of annexin V/7-AAD double-negative cells are demonstrated. (**D**) Western blot analysis to determine APAF1 knockdown and knockout efficacy in HT1080 cells. (**E**) APAF1 was knocked down in HT1080 control and *APAF1* knockout cells before treating them with erastin. 7-AAD and annexin V were read out by FACS. Primary FACS plots and respective quantifications of annexin V/7-AAD double-negative cells are demonstrated. (**F**) APAF1 was knocked down in HT1080 control and *APAF1* knockout cells before treating them with RSL3. 7-AAD and annexin V were read out by FACS. Primary FACS plots and respective quantifications of annexin V/7-AAD double-negative cells are demonstrated. The graphs show means ± SD. Statistical analysis was performed using Student’s *t* test. *P ≤ 0.05, **P ≤ 0.01, ***P ≤ 0.001.

To investigate the possibility of a scaffolding function of caspase-9 that may regulate ferroptosis sensitivity, we used the CRISPR-Cas9 technology to knock out the gene of *caspase-9*. The knockout was confirmed by Western blot (Fig. 3A). The *caspase-9* knockout cells were not sensitized to erastin- or RSL3-induced ferroptosis when compared to the parental cells, but when the crKO cells were treated with siRNA, a prominent sensitization to ferroptosis was consistently observed (Fig. 3, B and C). We confirmed the off-target siRNA-mediated effect in *APAF-1*-crKO HT1080 cells by detection of a similar pattern (Fig. 3, D to F). These data ultimately excluded the role of caspase-9 or APAF-1 in sensitizing HT1080 cells to ferroptosis. Finally, we excluded that the effect was mediated by transfection reagents or in an siRNA provider-dependent manner (figs. S3 to S5). We therefore focused on siRNA as the sensitizing mechanism, not as the target knockdown.

**Fig. 4.**
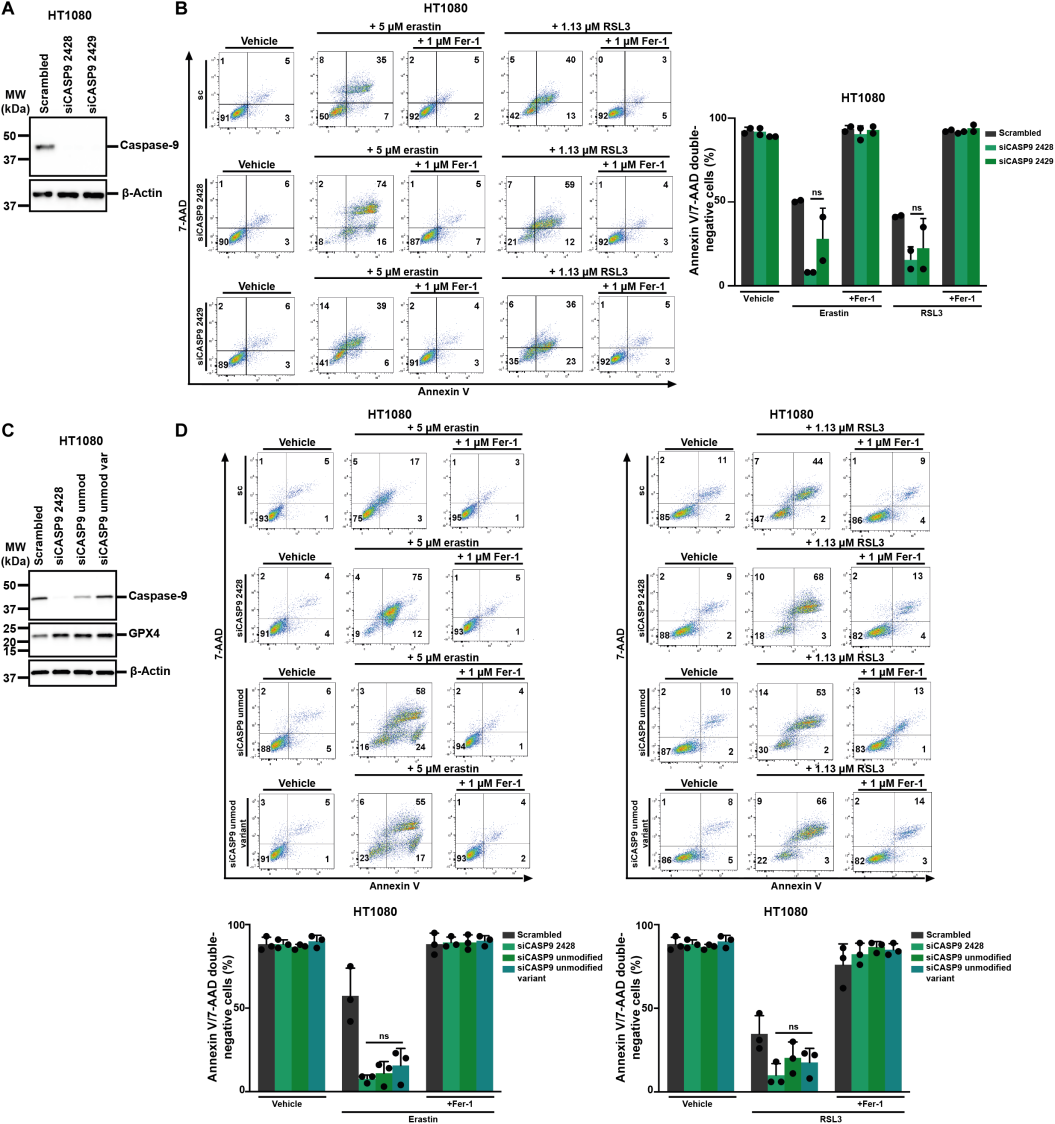
Different siRNAs targeting caspase-9 function to sensitize toward ferroptosis. (**A**) Western blot analysis to determine caspase-9 knockdown efficacy of different siRNAs in HT1080 cells. (**B**) Caspase-9 was knocked down in HT1080 cells using different siRNAs before treating them with erastin or RSL3. 7-AAD and annexin V were read out by FACS. Primary FACS plots and respective quantifications of annexin V/7-AAD double-negative cells are demonstrated. (**C**) Western blot analysis to determine caspase-9 knockdown efficacy and GPX4 expression after treatment with different siRNAs against caspase-9. (**D**) HT1080 cells were treated with siRNA against caspase-9 with locked nucleic acids (LNAs) (siCASP2428), one with the same sequence without LNAs (siCASP9 unmodified) or a variant where two nucleic acids were switched (siCASP9 unmodified variant) before induction of ferroptosis using erastin or RSL3. Primary FACS plots and respective quantifications of annexin V/7-AAD double-negative cells are demonstrated. The graphs show means ± SD. Statistical analysis was performed using Student’s *t* test or one-way ANOVA. ns, not significant.

The siRNA siCASP9-2428, which we used in all experiments up to now, was designed to target caspase-9 with locked nucleic acids (LNAs). We next investigated a different LNA-containing siRNA (siCASP9-2429), confirmed the knockdown efficacy (Fig. 4A), and again detected sensitization to ferroptosis induced by erastin or RSL3 (Fig. 4B). We then investigated siCASP9-2428 alongside an siRNA of identical sequence without LNAs (siCASP9-unmodified, siCASP9-umod) and a variant in which two nucleic acids were switched (siCASP-9-umod-variant). While siCASP9-umod and siCASP9-umod-variant, as expected, did not result in a substantial knockdown of the target gene (Fig. 4C), they did sensitize to erastin- and RSL3-induced ferroptosis, respectively (Fig. 4D). We noted that all siRNAs resulted in increased expression of GPX4 (Fig. 4C). Finally, we treated HT1080 cells with siCASP9-2428 or either single-stranded RNA of only the sense or the antisense strand before induction of ferroptosis by erastin or RSL3. In contrast to siCASP9-2428, the single-stranded RNAs failed to sensitize to ferroptosis (fig. S6). These data confirm potent ferroptosis sensitization by LNAs containing siRNAs while non–LNA-containing siRNAs are less potent. All of the single-stranded versions of RNA that we tested did not sensitize to ferroptosis.

Following the notion that siRNA-induced sensitization to ferroptosis is independent of the target knockdown, we wondered whether it is specifically associated with siRNAs that target components of the apoptosome (caspase-9 and APAF-1). We therefore treated HT1080 cells with siRNAs against the BCL2 family member MCL1 and the receptor interacting protein kinase 2 (RIPK2). We confirmed the knockdown efficacy of MCL1 (fig. S7A) and detected siRNA-induced sensitization to ferroptosis induced by erastin and RSL3 (fig. S7, B and C). Similarly, RIPK2 knockdown also sensitized to erastin- and RSL3-induced ferroptosis (fig. S7, D to F). These data suggested that siRNAs commonly sensitize to ferroptosis.

**Fig. 5.**
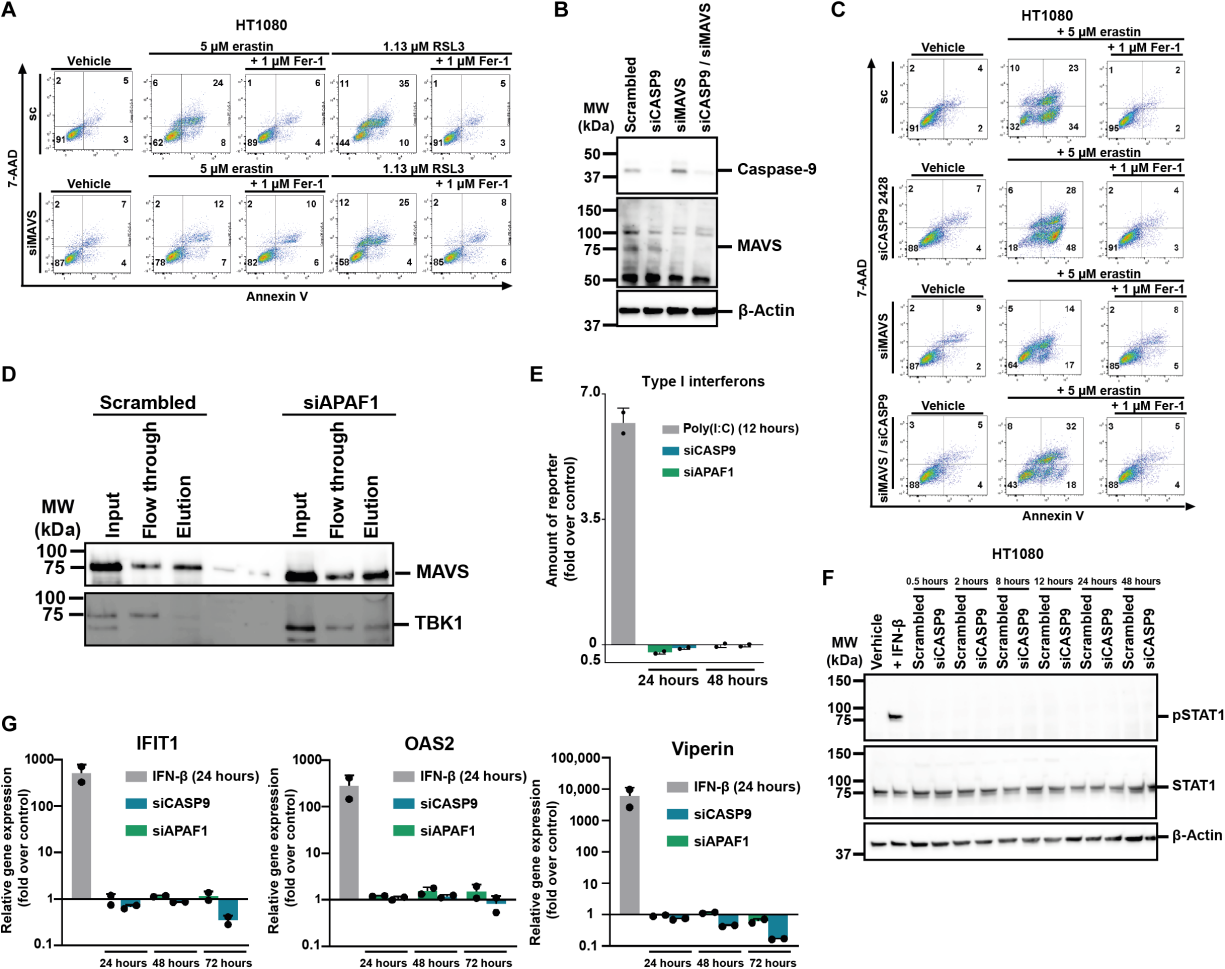
siRNAs against caspase-9 and APAF1 fail to activate canonical proinflammatory responses downstream of MAVS. (**A**) MAVS was knocked down in HT1080 cells before treating them with erastin or RSL3. 7-AAD and annexin V were read out by FACS. Primary FACS plots are demonstrated. (**B**) Western blot analysis to determine caspase-9 and MAVS knockdown efficacy in HT1080 cells. (**C**) Caspase-9, MAVS, or both were knocked down in HT1080 cells before treating them with erastin. 7-AAD and annexin V were read out by FACS. Primary FACS plots are demonstrated. (**D**) HT1080 cells were transfected with scrambled siRNA or siRNA against APAF1. After coimmunoprecipitation, MAVS was pulled down and the amount of MAVS and TBK1 in the different fractions was analyzed by Western blot. (**E**) Caspase-9 or APAF1 was knocked down in HT1080 cells before measuring type I interferons in the supernatant at indicated time points. Poly (I:C) was used as positive control. (**F**) Caspase-9 was knocked down in HT1080 cells before analyzing pSTAT1 protein levels at indicated time points. IFN-β was used as positive control. (**G**) Caspase-9 or APAF-1 was knocked down in HT1080 cells before analyzing mRNA levels of different interferon stimulated genes (ISGs) at indicated time points. IFN-β was used as positive control. IFIT1, interferon induced protein with tetratricopeptide repeats 1; IFN, interferon; IRF3, interferon regulatory factor 3; MAVS, mitochondrial antiviral-signaling protein; OAS2, 2′-5′-oligoadenylate synthetase 2; STAT, signal transducer and activator of transcription.

Given the finding that ssRNAs fail to sensitize to ferroptosis (fig. S6), we tested the possibility that siRNA can be sensed by the known RNA-sensing pathways to mediate the ferroptosis-sensitizing effect. Mitochondrial antiviral-signaling protein (MAVS) is known to be essential for effective antiviral immunity and senses dsRNA through integration of retinoic acid-inducible gene I–like receptor pathways, classically resulting in interferon responses. In contrast to the other siRNAs tested, knockdown of MAVS did not result in sensitization to ferroptosis (Fig. 5A). We therefore performed a double-knockdown experiment in which we added the MAVS-siRNA to the siCASP9-2428 and confirmed the knockdown efficacy of both proteins (Fig. 5B). siRNA against MAVS reversed the sensitizing effect of siCASP9-2428 (Fig. 5C). In similar double knockdown experiments with other sensitizing siRNAs, we again found that siRNA against MAVS reversed the sensitization of siRNAs against APAF-1 (fig. S8), MCL1 (fig. S9), and RIPK2 (fig. S10). These data suggested that MAVS is involved upstream of ferroptosis sensitization. We therefore investigated signaling pathways downstream of MAVS. We next tested whether siRNAs activate canonical MAVS signaling including TBK1 recruitment, interferon regulatory transcription factor 3 (IRF3), and IκBα phosphorylation and production of interferons. As expected, treatment with siRNA was associated with the recruitment of TBK1 to MAVS, as demonstrated by immunoprecipitation (Fig. 5D). However, siRNA treatment did not result in the production of type I interferons (Fig. 5E), downstream phosphorylation of signal transducer and activator of transcription 1 (STAT1) (Fig. 5F), or mRNA expression of the interferon stimulated genes *IFIT1, OAS2*, and *viperin* (Fig. 5G). In contrast to poly(I:C), which we used as positive control, siRNAs against caspase-9 or APAF-1 failed to induce the expression of tumor necrosis factor–α (TNF-α), interleukin-6 (IL-6), or IL-8 (fig. S11, A to C). Finally, we did not detect substantial levels of phosphorylation of IRF3 or IκBα (fig. S11, D and E). These data indicate that MAVS does not regulate ferroptosis sensitivity in a classical way.

**Fig. 6.**
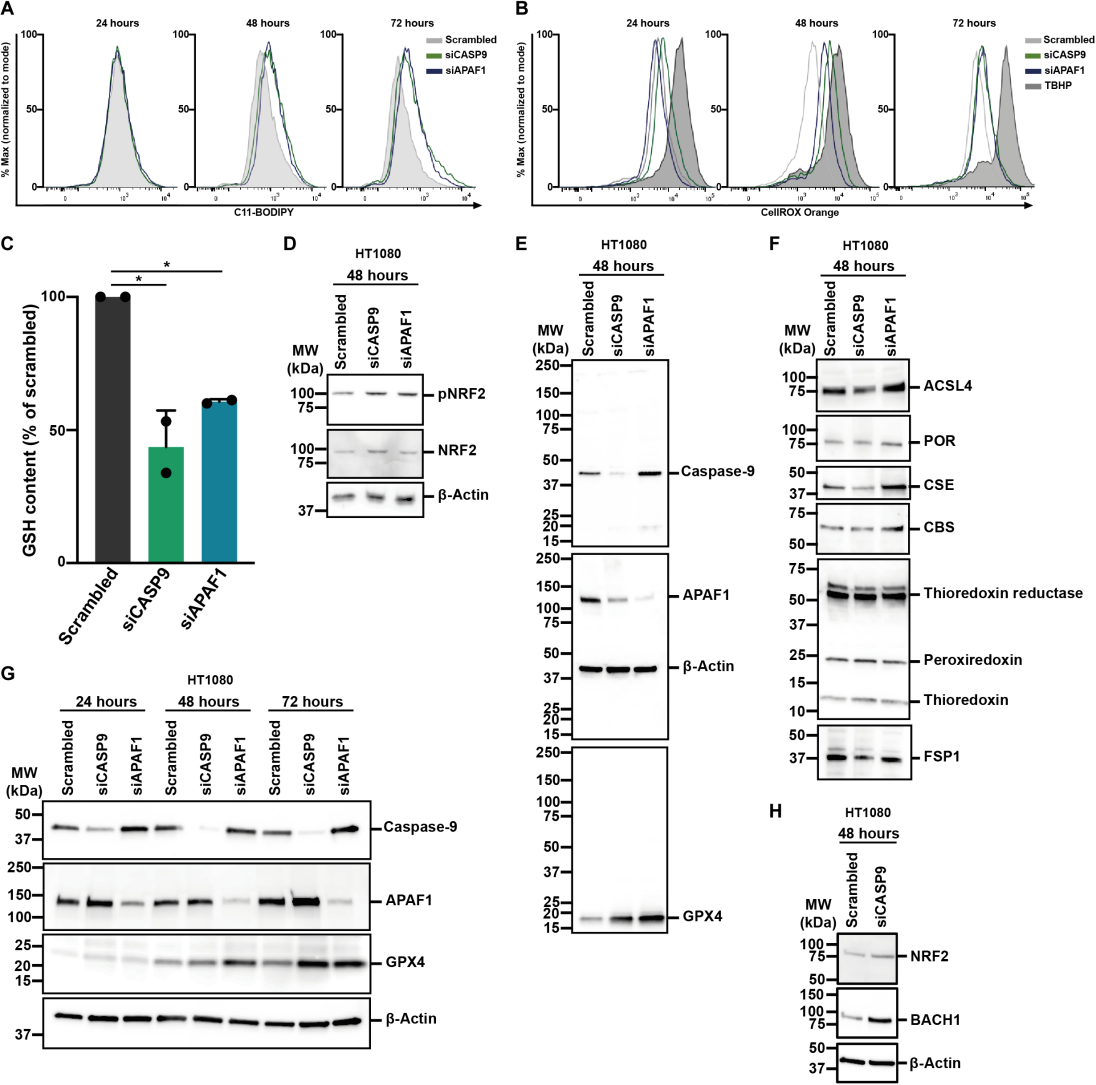
siRNA transfection increases ferroptosis sensitivity through GPX4 and glutathione regulation. (**A**) Caspase-9 or APAF1 was knocked down in HT1080 cells before measuring C-11 BODIPY as a marker for lipid peroxidation at indicated time points. (**B**) Caspase-9 or APAF1 was knocked down in HT1080 cells before measuring reactive oxygen species (ROS) at indicated time points. TBHP (*tert*-butyl hydroperoxide) was used as positive control. (**C**) Caspase-9 or APAF1 was knocked down in HT1080 cells before measuring glutathione (GSH) levels after 48 hours. (**D**) Caspase-9 or APAF1 was knocked down in HT1080 cells and expression of pNRF2 protein was analyzed after 48 hours. (**E**) Caspase-9 or APAF1 was knocked down in HT1080 cells and GPX4 protein expression was analyzed after 48 hours. (**F**) In the cell lysates presented in (D), expression of key ferroptosis proteins was analyzed after 48 hours. (**G**) Caspase-9 or APAF1 was knocked down in HT1080 cells and expression of GPX4 protein was analyzed at indicated time points. (**H**) Caspase-9 was silenced in HT1080 cells and expression of NRF2 and BACH1 protein was analyzed after 48 hours. The graphs show means ± SD. Statistical analysis was performed using Student’s *t* test. *P ≤ 0.05. ACSL4, acyl-CoA synthetase long-chain family member 4; BACH1, BTB domain and CNC homolog 1; CBS, cystathionine beta-synthase; CSE, cystathionine gamma lyase; FSP1, AIFM2, ferroptosis suppressor protein 1; GPX4, glutathione peroxidase 4; GSH, glutathione; NRF2, nuclear factor erythroid 2-related factor 2; POR, cytochrome p450 oxidoreductase; TBHP, *tert*-butyl hydroperoxide.

**Fig. 7.**
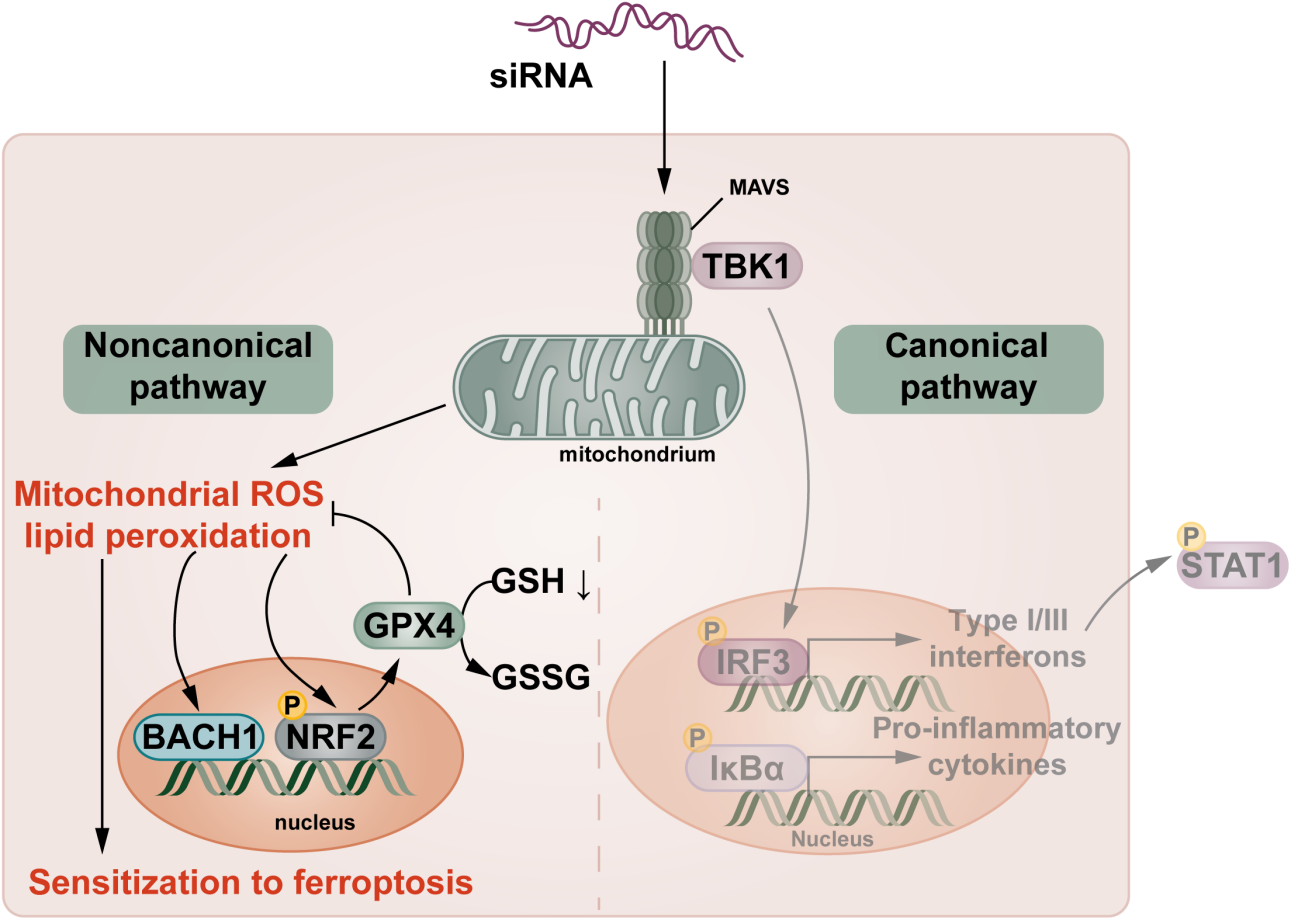
Concept of siRNA-mediated noncanonical MAVS signaling. siRNA treatment sensitizes to ferroptosis in a MAVS-dependent manner that involves increases in mitochondrial production of reactive oxygen species, lipid peroxidation, BACH1 expression, Nrf2 phosphorylation, GPX4 up-regulation, and GSH depletion (noncanonical signaling). In contrast, the canonical pathway downstream of MAVS that includes phosphorylation of IRF3, IκBα, the production of interferons, and other proinflammatory cytokines as well as downstream phosphorylation of STAT1 is not activated.

To better understand the nature of siRNA-induced ferroptosis sensitization, we treated HT1080 cells with siRNAs against caspase-9 or APAF-1 and assessed lipid peroxidation by C11-BODIPY over time without induction of ferroptosis. As demonstrated in Fig. 6A, both siRNAs resulted in an increase of the C11-BODIPY signal at the 48-hour and the 72-hour time points. In a separate experiment, we measured reactive oxygen species (ROS) using the CellROX Orange assay over time. While no effect was detected at the 24-hour value, the 48-hour and 72-hour time points exhibited an increase in CellROX Orange in the cells treated with siRNA (Fig. 6B). TBHP (*tert*-butyl hydroperoxide) was used as positive control. Given the nonspecificity of glutathione (GSH) plate reader assays, we used liquid chromatography–mass spectrometry (LC-MS) to detect intracellular GSH levels. Remarkably, treating HT1080 cells with siRNAs resulted in an approximately 50% decrease in intracellular GSH levels 48 hours following transfection (Fig. 6C). As expected, the increase in GPX4 expression was associated with a slight increase in phosphorylation of the transcription factor NRF2 (Fig. 6D). In contrast, however, the expression of most classical NRF2 target genes was not up-regulated (fig. S12). At the same time point, we detected a fundamental up-regulation of GPX4 (Fig. 6E). The same lysates were additionally investigated to assess expression of ferroptosis-associated proteins. Expression levels of acyl-CoA synthetase long-chain family member 4 (ACSL4), cytochrome P450 oxidoreductase POR, the transsulfuration pathway proteins cystathionine beta synthase (CBS) and cystathionine gamma lyase (CSE), thioredoxin, peroxiredoxin, thioredoxin reductase, and ferroptosis-suppressor protein 1 (FSP1, also referred to as AIFM2) did not consistently change upon siRNA transfection (Fig. 6F). In contrast, Fig. 6G demonstrates the consistent up-regulation of GPX4 in HT1080 cells over time. Of note, the protein expression of the transcription factor BACH1, which regulates NRF2 activation and ferroptosis sensitivity (*22*, *23*), increased more substantially compared to NRF2, resulting in an increased BACH1/NRF2 ratio (Fig. 6H). Collectively, the increase in ROS and lipid peroxidation associated with a positive BACH1/pNRF2 ratio drives the up-regulation of GPX4, which may explain GSH depletion and indicates that siRNAs lower the threshold for cellular ferroptosis (Fig. 7).

## DISCUSSION

Several publications have used the technology of siRNA-mediated knockdown of various target proteins, such as prominin 2 (*24*), or other proteins to investigate cell death, and reported sensitization to ferroptosis (*25*–*29*). The data presented here do not contradict any of these data, but demonstrate that treatment with siRNAs on its own can cause sensitization to ferroptosis independent of the intended siRNA target.

The detection of off-target siRNA effects reported in this study was somewhat serendipitous, occurring when our parental cells were sensitized to ferroptosis upon caspase-9 knockdown but not by the CRISPR-Cas9–mediated *caspase-9* knockout. The knockout cells, however, were sensitized by the siRNA without carrying its default target. It is not surprising that MAVS, the central RNA integration hub (*30*–*32*), mediates this effect, although through a noncanonical pathway that does not involve NF-kB or IRF3 signaling. The expression of MAVS at the outer mitochondrial membrane suggests a direct effect on mitochondria as the primary source of ROS that drive BACH1/NRF2-dependent GPX4 expression and sensitization to ferroptosis. The precise mechanisms of GPX4 up-regulation, however, may not be entirely Nrf2-dependent and BACH1-controlled.

Untoward outcomes of kidney transplantation include acute tubular necrosis and acute kidney injury. Attempts to target key mediators of kidney injury by siRNA, such as p53, have failed in clinical trials, which have been withdrawn for unknown or at least unpublished reasons. These trials tested synthetic double-stranded siRNA oligonucleotides directed against p53 mRNA (QPI-1002) (*33*) for the prevention of delayed graft function after renal transplantation. In another approach, the siRNA target was a complement factor (C3) for the treatment of membranous nephropathy (*34*). Therein, all siRNA-treated mice exhibited much higher values of proteinuria compared with experiments in which the target protein was genetically knocked out (*34*). Many other siRNA-based approaches are currently being followed, (*35*) and recently, five siRNA-based therapeutics have obtained U.S. Food and Drug Administration approval (*36*), a prominent example of which is the PCSK9 targeting drug inclisiran (*37*, *38*). In our study, we did not formally test ferroptosis-driven side effects in any in vivo model system, but at the current stage of research, we cannot exclude that severe adverse events may be aggravated following siRNA treatment.

In conclusion, we identify a MAVS-dependent mechanism of siRNA recognition that lowers the threshold to ferroptosis. The findings highlight the importance to not exclusively rely on individual silencing approaches in biomedical research and favor additional testing such as CRISPR-Cas9–mediated knockout for target validation, especially when cell death readout systems are used.

## MATERIALS AND METHODS

### Cell lines and cell culture

Human HT1080 and HEK293T cells were purchased from the American Type Culture Collection and cultured in Dulbecco’s modified Eagle’s medium (DMEM) supplemented with 10% (v/v) fetal bovine serum (FBS) (Thermo Fisher Scientific), penicillin (100 U/ml), and streptomycin (100 μg/ml). CD10 cells were kindly provided by R. Kramann and cultured in DMEM/F12 supplemented with 10% (v/v) FBS (Thermo Fisher Scientific), penicillin (100 U/ml), and streptomycin (100 μg/ml). Human embryonic kidney (HEK)-Blue IFN-α/β cells were kindly provided by Axel Roers, and cultured DMEM was supplemented with 10% (v/v) FBS, blasticidin (30 μg/ml), Zeocin (100 μg/ml), Normocin (100 μg/ml), penicillin (100 U/ml), and streptomycin (100 μg/ml). All cells were cultured in a humidified 5% CO_2_ atmosphere.

### siRNA-mediated knockdown

HT1080 cells were plated in a 10-cm petri dish in 15 ml of antibiotic-free medium. After 24 hours, 180 pmol siRNA and 24 μl of Lipofectamine (Thermo Fisher Scientific) were each mixed in 1.5 ml of Opti-MEM I Medium (Thermo Fisher Scientific) without serum, combined and incubated at room temperature for 20 min before dropping the mixture on the cells. The following day, cells were harvested, plated into six-well plates and experiments were performed as described below. Knockdown efficacy was determined by protein level.

### Generation of HT1080 knockout cells

The LentiCRISPRv2 blast vectors containing guides targeting the human caspase-9 (CGCAGCAGTCCAGAGCACCG) or human APAF1 gene (ACAGCCTGCCATTCCATGTA) were provided by Stephen Tait. HEK293T cells were transfected with LentiCRISPRv2 blast vectors and packing plasmids (pspax2, pMD2.G) using ViaFect transfection reagent and the medium was changed the next day. Forty-eight hours after transfection, HT1080 cells were transduced with viral supernatant and then selected using blasticidin (5 μg/ml). Knockout efficacy was determined by protein level in polyclonal cells.

### Western blotting

Cells were lysed in ice-cold 50 mM tris-HCl, pH 7.5, 150 mM NaCl, 1% NP-40, and 5 mM EDTA (NP-40 Buffer) or 50 mM tris-HCl, pH 8, 150 mM NaCl, 1% NP-40, 0.5% sodium deoxycholate, and 0.1% sodium dodecyl sulfate (SDS) (radioimmunoprecipitation assay buffer) supplemented with PhosSTOP (Merck), cOmplete (Merck), and 1 mM phenylmethylsulfonyl fluoride for 30 min on ice. Insoluble material was removed by centrifugation (14,000*g*, 30 min, 4°C). Protein concentration was determined using a commercial BCA assay kit according to the manufacturer’s instructions. Equal amounts of protein (typically 25 μg per lane) were resolved on a 4 to 15% gradient SDS–polyacrylamide gel electrophoresis gel and transferred to a polyvinylidene difluoride membrane (Bio-Rad). After blocking for 1 hour at room temperature, primary antibody incubation was performed at 4°C overnight. Secondary antibodies were applied at concentrations of 1:5,000. Proteins were then visualized by enhanced chemiluminescence (ECL).

### Quantitative real-time PCR

RNA was isolated using the RNeasy Plus Mini Kit according to the manufacturer’s instructions and transcribed into cDNA using the High-Capacity cDNA Reverse Transcription Kit. cDNA MasterMix (10 μl) containing 2 μl of 10× RT Buffer, 0.8 μl of 25× dNTP Mix, 2 μl of Random Primers, 1 μl of MultiScribe Reverse Transcriptase, and 1 μl of RNase Inhibitor was added to 10 μl of water containing 2 μg of RNA. Reaction was performed as follows: 25°C for 10 min, 37°C for 2 hours, 85°C for 5 min, 4°C forever. The cDNA was diluted to 5 ng/μl and 2.5 μl of this dilution was added to 10 μl of qPCR Mastermix containing 6.25 μl of PowerUP SYBR Green and 0.5 μM of each primer or 1 μl of 1× QuantiTect Primer Assay (Qiagen). Reaction was performed on the CFX Connect (Bio-Rad) as follows: 95°C for 3 min; 40 cycles: 95°C for 10 s, 57°C for 10 s, 72°C for 30 s, and 95°C for 10 s. Each sample was measured in duplicate or triplicate and relative gene expression of housekeeping genes was determined using the 2^−ΔΔCt^ method. For reagents, see Table 1, and for primers, see Table 2 and 3.

**1.** Details of reagents used.

Reagent or resource	Source	Identifier
Antibodies		
Recombinant anti-caspase-9 antibody [EPR18107]	Abcam	ab202068
Recombinant anti-APAF1 antibody [EPR21112-102]	Abcam	ab234436
Anti-FACL4 antibody [EPR8640]	Abcam	ab155282
PRX pathway (TRX, TXNRD1, PRX1) WB cocktail	Abcam	ab184868
CBS monoclonal antibody (GT519)	Thermo Fisher Scientific	MA5-17273
Anti-gamma cystationase mouse monoclonal antibody	Proteintech	60234-1-Ig
Anti-glutathione peroxidase 4 antibody [EPNCIR144]	Abcam	ab125066
Recombinant anti-cytochrome P450 reductase antibody [EPR14479(B)]	Abcam	ab180597
Anti-human FSP1 antibody	Pedro Friedmann Angeli	n/a
Phospho-Stat1 (Tyr701) (58D6)	Cell Signaling	9167S
Stat1 antibody	Cell Signaling	9172S
Phospho-IκBα (Ser32) (14D4)	Cell Signaling	2859S
IκBα (44D4)	Cell Signaling	4812S
Phospho-IRF-3 (Ser396) (4D4G)	Cell Signaling	4947S
IRF-3 (D6I4C) XP	Cell Signaling	11904S
MAVS antibody	Cell Signaling	3993S
RIPK2	Cell Signaling	3493
MCL1	Abcam	ab32087
HMO-1	Abcam	ab68477
GCLC	Abcam	ab190685
GCLM	Abcam	ab126704
GSTA1	Proteintech	14475-1-AP
BACH1 antibody (F9)	Santa Cruz Biotechnology	sc-271211
β-Actin	Cell Signaling	3700S
Anti-mouse IgG; HRP-linked antibody	Cell Signaling	7076S
Anti-rabbit IgG; HRP-linked antibody	Cell Signaling	7074S
Compounds and chemicals		
DMEM, high glucose, pyruvate	Thermo Fisher Scientific	41966029
Fetal bovine serum	Thermo Fisher Scientific	10270106
Penicillin-streptomycin	Thermo Fisher Scientific	15140122
Blasticidin	InvivoGen	ant-bl-1
Zeocin	InvivoGen	ant-zn-05
Normocin	InvivoGen	ant-nr-1
Erastin (type 1 FIN)	Sigma Aldrich	E7781
RSL3 (type 2 FIN)	Selleck Chemicals	S8155
FIN56 (type 3 FIN)	Cayman Chemical	Cay25180
FINO2 (type 4 FIN)	Cayman Chemical	Cay25096
Ferrostatin-1 (Fer-1)	Merck Millipore	341494
Deferoxamine mesylate (DFO)	Selleck Chemicals	S5742
Liproxstatin-1 (LPX)	Sigma-Aldrich	SML1414
zVAD fmk	BD Biosciences	550377
Emricasan	Sigma-Aldrich	SML2227
Q-VD-Oph	Selleck Chemicals	S7311
7-Cl-O-Nec-1 (Nec-1s)	Merck Millipore	5.04297.0001
S63845	Selleck Chemicals	S8383
Navitoclax (ABT-263)	Selleck Chemicals	S1001
Human TNF-α	BioLegend	570108
Recombinant human IFN-β	Peprotech	300-02BC
Poly(I:C)	InvivoGen	tlrl-pic
7-AAD	BD Biosciences	559925
Annexin-V–FITC	BD Biosciences	556420
Annexin-V binding buffer	BD Biosciences	556454
BODIPY 581/591 C11	Thermo Fisher Scientific	D3861
CellROX Orange Flow Cytometry Assay Kit	Thermo Fisher Scientific	C10493
Bradford assay	Thermo Fisher Scientific	23225
ECL prime Western blotting system	Thermo Fisher Scientific	GERPN2232
Opti-MEM I medium	Thermo Fisher Scientific	31985062
Lipofectamine RNAiMAX	Thermo Fisher Scientific	13778075
Human caspase-9 siRNA (s2428) LOT: ASO2JQOD	Thermo Fisher Scientific	4392421
Human caspase-9 siRNA (s2428) LOT: ASO2KO10	Thermo Fisher Scientific	4392421
Human caspase-9 siRNA (s2428) LOT: ASO2K01N	Thermo Fisher Scientific	4392421
Human caspase-9 siRNA (s2428) LOT: ASO2JEFTT	Thermo Fisher Scientific	4392421
Human caspase-9 siRNA (s2429)	Thermo Fisher Scientific	4392421
Human APAF1 siRNA (s1413)	Thermo Fisher Scientific	4392421
Human MCL1 siRNA (s8583)	Thermo Fisher Scientific	4392421
Human RIPK2 siRNA (s247)	Thermo Fisher Scientific	4392421
Human MAVS siRNA (s33178)	Thermo Fisher Scientific	4392421
Human NRF2 siRNA (s9491)	Thermo Fisher Scientific	4392421
Human NRF2 siRNA (s9493)	Thermo Fisher Scientific	4392421
Human caspase-9 siRNA	Eurofins	CGGUGAAAGGGAUUUAUAATT
Human caspase-9 siRNA variant	Eurofins	CGGU**AG**AAGGGAUUUAUAATT
Human caspase-9 siRNA single-strand sense	IDT	CGGUGAAAGGGAUUUAUAAtt (tt: locked nucleic acids)
Human caspase-9 siRNA single-strand antisense	IDT	UUAUAAAUCCCUUUCACCGaa (aa: locked nucleic acids)
Silencer select negative control #1 siRNA	Thermo Fisher Scientific	4390843
ViaFect transfection reagent	Promega	E4981
High-Capacity cDNA Reverse Transcription Kit	Thermo Fisher Scientific	4368814
RNase inhibitor	Thermo Fisher Scientific	N8080119
PowerUp SYBR Green Master Mix	Thermo Fisher Scientific	A25776
Caspase 9 Colorimetric Activity Assay Kit	Merck Millipore	APT139
RNeasy Plus Mini Kit	Qiagen	74134
QUANTI-Blue Solution	InvivoGen	rep-qbs
Elecsys IL-6	Roche Diagnostics	09015612190
TNF-α IMMULITE (LKNF1)	Siemens	6602826
Interleukin-8 IMMULITE (LK8P1)	Siemens	6604136

**Table 2.** Sequences of designed qPCR primers.

Gene	Forward primer	Reverse primer
IFIT1	GAGGAGCCTGGCTAAGCAAA	GCTCCAGACTATCCTTGACCTG
OAS2	AACACCATCTGTGACGTCCT	TGTTTTCCGTCCATAGGAGCC
Viperin	ACCCCAACCAGCGTCAACTAT	TGAAGAAATGGCTCTCCACCTG
β-actin	AGCCTCGCCTTTGCCGAT	CTGACCCATGCCCACCATCA
GAPDH	GTTCGACAGTCAGCCGCATC	GGCGCCCAATACGACCAAAT
b2m	AAGTGGGATCGAGACATGTAAGCA	GGAATTCATCCAATCCAAATGCGG

**Table 3.** Details of commercial qPCR primers.

Gene	QuantiTect assay	Source	Identifier
HMO-1	Hs_HMOX_1_SG	Qiagen	QT00092645
SLC7A11	Hs_SLC7A11_1_SG	Qiagen	QT00002674
GCLC	Hs_GCLC_1_SG	Qiagen	QT00037310
GCLM	Hs_GCLM_1_SG	Qiagen	QT00038710

### Cell death assays

Cell death assays were performed in six-well plates. Ferroptosis was induced using established FINs: Type 1 FIN: erastin, type 2 FIN: RSL3, type 3 FIN: FIN56, type 4 FIN: FINO2. Unless otherwise indicated, we used 5 μM erastin, 1.13 μM RSL3, 10 μM FIN56, or 10 μM FINO2. Apoptosis was induced by the addition of 5 μM navitoclax and 5 μM of the MCL-1 inhibitor S63845. After the indicated time points, cells were collected and prepared for flow cytometry. Cells were harvested and the pellets were washed twice in phosphate-buffered saline (PBS) and stained with 5 μl of 7-AAD and 5 μl of annexin-V–FITC (fluorescein isothiocyanate) added to 100 μl of annexin-V binding buffer. After 15 min, cells were recorded on the LSR Fortessa with the FACS Diva v9.0.1 software (BD Biosciences) and subsequently analyzed using FlowJo v10 software (Tree Star). The flow cytometry procedure was supported by the Flow Cytometry Core Facility of the CMCB Technology Platform at TU Dresden.

### BODIPY 581/591 C11 analysis

To assess lipid peroxidation, cells were harvested and stained with 2 μM BODIPY 581/591 C11 in 500 μl of Hanks’ balanced salt solution (HBSS) for 10 min at 37°C. Subsequently, cells were washed twice with HBSS and analyzed by flow cytometry.

### Measurement of ROS

To assess ROS production, the CellROX Orange Flow Cytometry Assay Kit was used. For positive controls, cells were incubated with 300 μM TBHP for 1 hour at 37°C to induce ROS. Cells were then stained in six-well plates in complete growth medium using 750 nM CellROX Orange and incubated for 45 min at 37°C before harvesting them. Subsequently, cells were washed twice with PBS and analyzed by flow cytometry.

### Caspase-9 activity assay

Caspase-9 activity was measured using the Caspase 9 Colorimetric Activity Assay Kit. Cells were harvested and lysed using 1× Cell Lysis Buffer for 10 min on ice before centrifuging at 10,000*g* for 5 min. Equal amounts of protein (usually 200 μg) were diluted with deionized water to 70 μl and further analyzed by adding 20 μl of 5× Assay Buffer and 10 μl of caspase-9 substrate and incubated for 2 hours at 37°C. Optical density (OD) was measured at 405 nm and relative caspase-9 activity was calculated in relation to untreated control cells.

### Assessment of INF-α/β

Levels of INF-α/β (type I interferons) in the supernatant of HT1080 cells were determined using the reporter cell line HEK-Blue IFN-α/β. To this end 5 × 10^4^ HEK-Blue IFN-α/β cells were seeded in 100 μl of medium without blasticidin, Zeocin, or Normocin in 96-well plates. At the same time, 100 μl of the supernatant from HT1080 cells or medium only as control was added and incubated overnight at 37°C and 5% CO_2_. The next day, QUANTI-Blue Solution was prepared according to the manufacturer’s instructions by diluting QUANTI-Blue Reagent and QUANTI-Blue Buffer 1:100 in sterile water and incubating for 10 min at room temperature. QUANTI-Blue Solution (180 μl) was mixed with 20 μl of the supernatant from HEK-Blue IFN-α/β cells in a 96-well plate. After incubation for 1 hour at 37°C, the OD was measured at 620 nm using a microplate reader. The fold induction of type I interferons over control was calculated based on measured OD values.

### Assessment of IL-6, IL-8, and TNF-α

IL-6, IL-8, and TNF-α were measured in routine diagnostics at the Institute for Clinical Chemistry and Laboratory Medicine of the University Hospital Dresden using the Elecsys IL-6 Kit, the Interleukin-8 IMMULITE Kit, and the TNF-α IMMULITE Kit, respectively.

### Mass spectrometry (LC-ESI-QToF)

Cells were placed on ice and washed with 0.9% NaCl before covering them with −80°C cold quenching buffer (80% LC-MS–grade methanol) followed by 20-min incubation time at −80°C. Subsequently, cells were scraped and centrifuged at 14,000*g* at 4°C for 10 min. The supernatant was centrifuged at 14,000*g* at 4°C for 10 min again before freezing it at −80°C until further analysis. For mass spectrometry–based analysis using an Aquity I-class ultraperformance LC system (Waters) coupled to a high-resolution quadrupole time-of-flight mass spectrometer (QToF-MS) (Vion IMS QToF, Waters), 200-μl aliquots of the aforementioned cell lysates were dried down in a vacuum-assisted centrifuge for at least 2.5 hours and afterward reconstituted in 100 μl of initial mobile phase (10%/90% water/acetonitrile containing 0.1% formic acid). For chromatographic separation, a Waters Acquity UPLC BEH Column (2.1 × 100 mm, 1.7 μm) at 45°C and a gradient of mobile phases A (water/0.1% formic acid) and B (acetonitrile/0.1% formic acid) were used. Calibrators and samples (7.5 μl, kept at 4°C in the autosampler, were injected into the LC-QToF-MS system at a flow rate of 0.4 ml/min. Directly after injection at 10% mobile phase A, proportions of A linearly increased to 20% at 2.0 min, to 48% at 4.0 min, and further to 90% at 5.0 min. After a hold for 1 min at 90% mobile phase A, the gradient returned back to initial conditions at 6.3 min, followed by another 1.2 min for column re-equilibration. For identification and quantification of GSH, a single standard of GSH was purchased from Sigma-Aldrich (Munich, Germany). After desolvation and respective dilution in the mobile phase, the GSH standard was injected into the LC-QToF-MS to determine analyte-specific characteristics, such as LC retention time (3.30 min), accurate mass ([M + H^+^] = 308.0915 Da) and the collision cross-section (CCS) value (168.06 Å^2^), a metabolite specific measure of ion mobility. GSH in calibrators and samples was detected by using the high-definition MS^E^ data acquisition mode that includes ion mobility separation and determination of CCS data, and accurate precursor and respective fragment ion masses of all ions. Positive electrospray ionization source parameters were set to 1.0 kV for capillary voltage, to 120°C and 550°C for source and desolvation temperatures, respectively, as well as to the respective cone- and desolvation gas flows at 50 and 900 liters/hour. Data were evaluated using Waters with UNIFI Software package (Version 1.9.4.053).

### Statistical analysis

Statistical analyses were performed with Prism 9 (GraphPad software, San Diego, CA, USA) using Student’s *t* test for two groups or one-way analysis of variance (ANOVA) for three and more groups. Data were considered significant when *P ≤ 0.05, **P ≤ 0.01, or ***P ≤ 0.001.
