# Supplementary material for: Treatment with siRNAs is commonly associated with GPX4 up-regulation and target knockdown-independent sensitization to ferroptosis
Source: Sci Adv. 2024 Mar 15;10(11):eadk7329. doi: 10.1126/sciadv.adk7329 (PMC10942120; doi:10.1126/sciadv.adk7329)
Supplement: Supplementary file 1 — Figs. S1 to S12 [file sciadv.adk7329_sm.pdf]

Science Advances
AAAS

Supplementary Materials for

Treatment with siRNAs is commonly associated with GPX4 up-regulation and target knockdown-independent sensitization to ferroptosis

Anne von Mässenhausen *et al.*

Corresponding author: Andreas Linkermann, andreas.linkermann@ukdd.de

Sci. Adv. **10**, eadk7329 (2024)
DOI: 10.1126/sciadv.adk7329

This PDF file includes:

Figs. S1 to S12

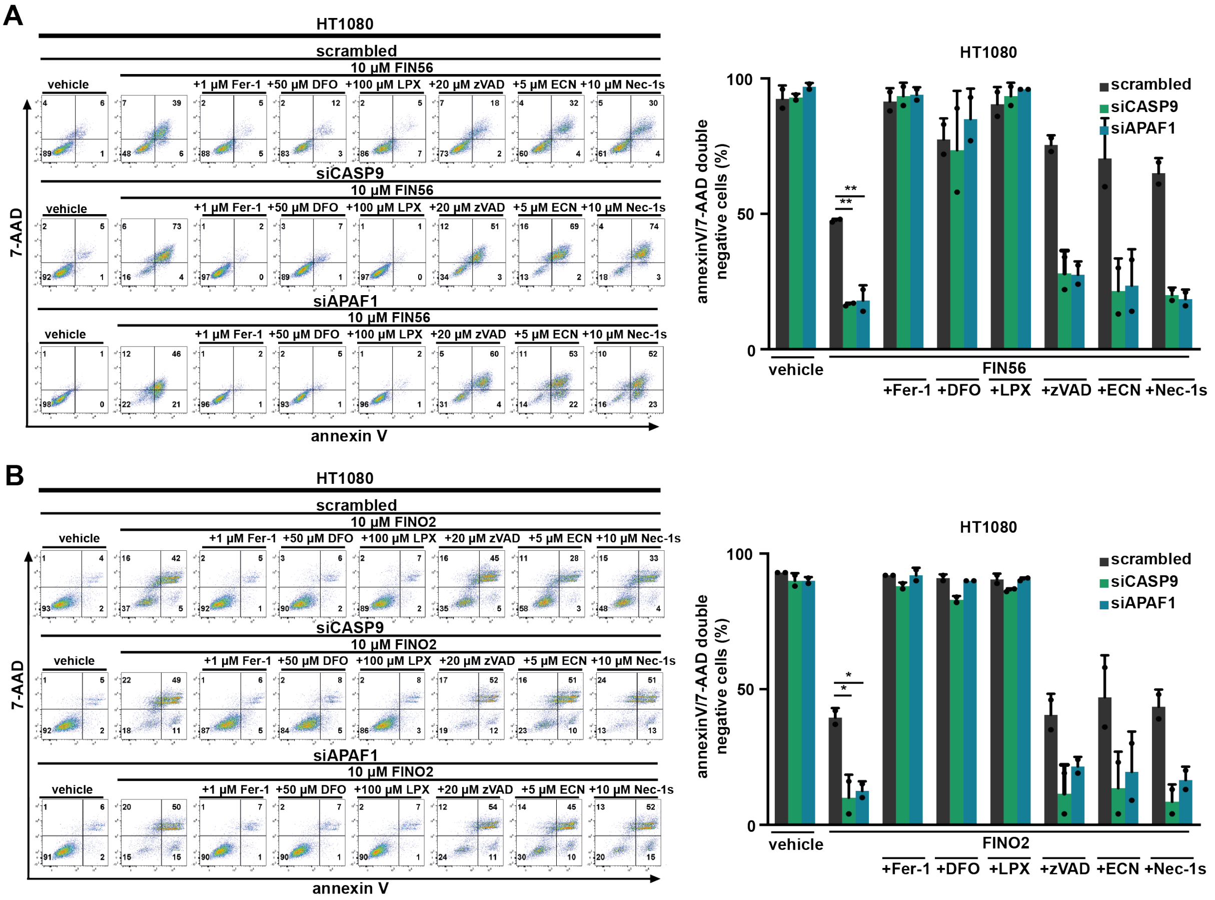

Figure S1. siRNA against caspase-9 and APAF1 sensitize toward ferroptosis. A) Caspase-9 or APAF1 was knocked down in HT1080 cells before treating them with FIN56. 7-AAD and annexin V were read out by FACS. Primary FACS plots and respective quantifications of annexin V/7-AAD double negative cells are demonstrated. **B**) Caspase-9 or APAF1 was knocked down in HT1080 cells before treating them with FINO2. 7-AAD and annexin V were read out by FACS. Primary FACS plots and respective quantifications of annexin V/7-AAD double negative cells are demonstrated. The graphs show means ± SD. Statistical analysis was performed using one way ANOVA. *p ≤ 0.05, **p ≤ 0.01. DFO: deferoxamine, LPX: liproxstatin, ECN: emricasan.

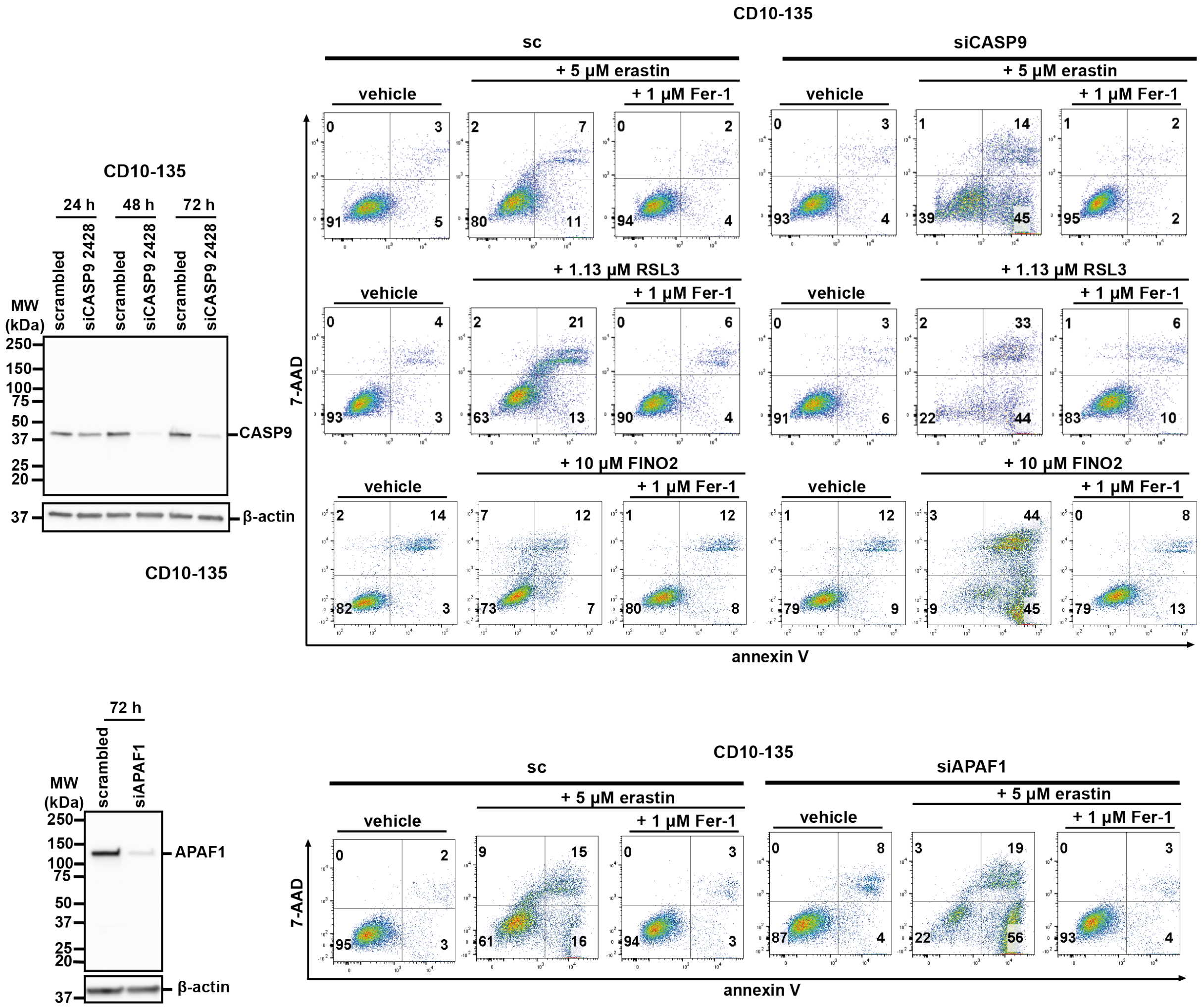

Figure S2. siRNA against caspase-9 and APAF1 sensitize CD10-135 cells toward ferroptosis. CD10-135 cells were treated with siRNA against caspase-9 or APAF-1 before induction of ferroptosis using erastin or RSL3. Primary FACS plots are demonstrated. Corresponding Western blot analysis to determine knockdown efficacy of caspase-9 and APAF1 at indicated time points after siRNA treatment are shown on the left.

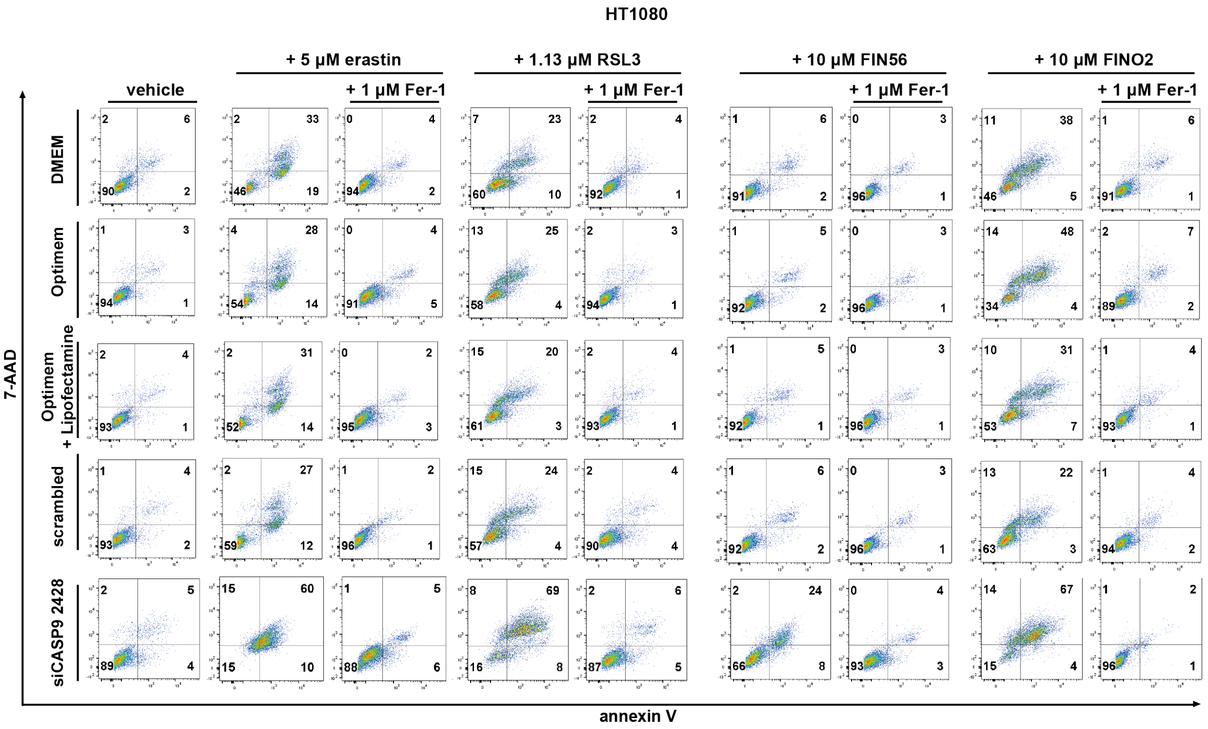

Figure S3. Transfection reagents do not exhibit sensitization toward ferroptosis. HT1080 cells were treated with culture medium (DMEM) or components of the siRNA transfection protocol without addition of siRNA (Optimem and Optimem + Lipofectamine), or with scrambled siRNA or siRNA against caspase-9 as a control before induction of ferroptosis using erastin, RSL3, FIN56 or FINO2. Primary FACS plots are demonstrated.

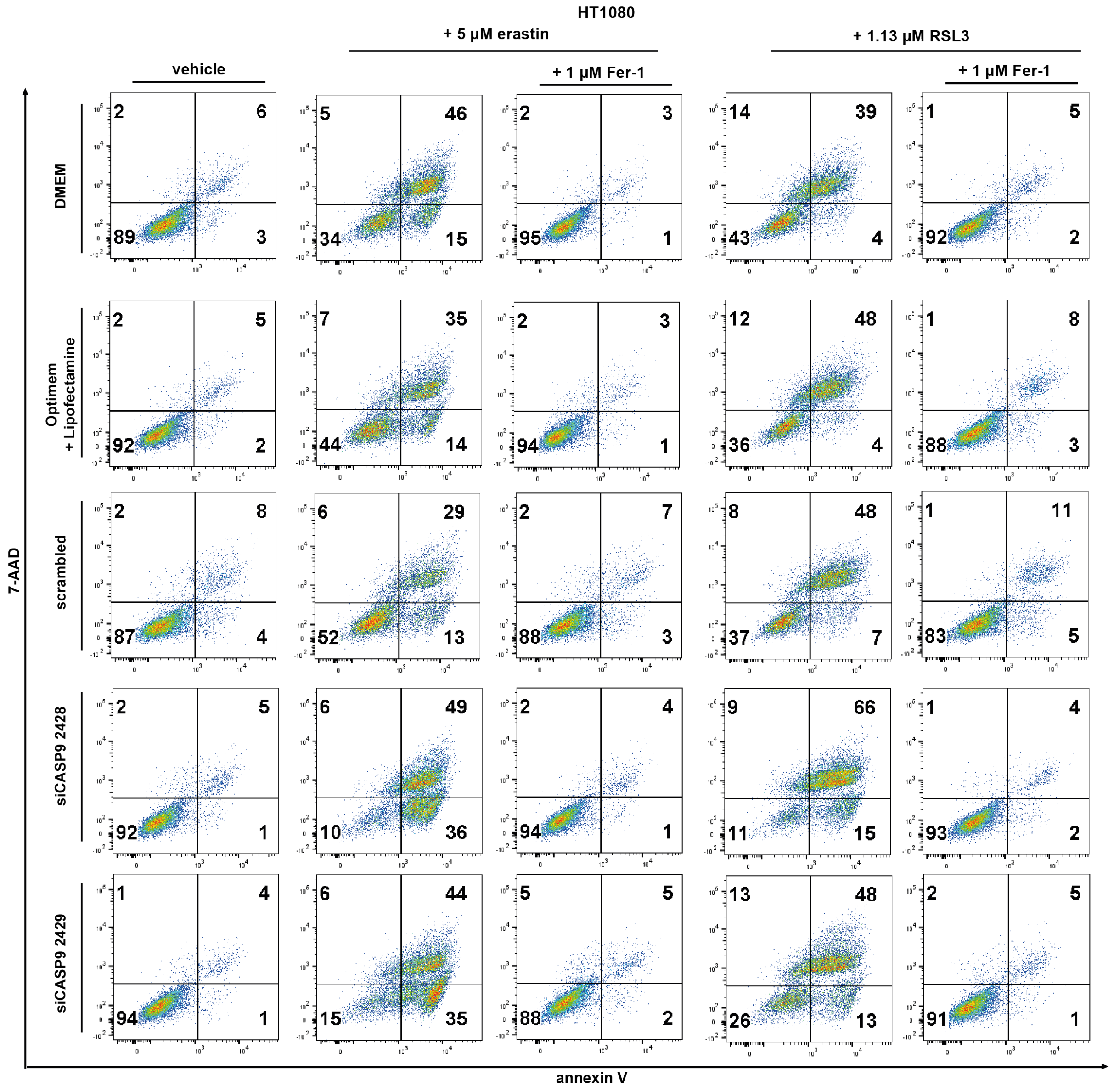

Figure S4. Transfection reagents do not exhibit sensitization toward ferroptosis. HT1080 cells were treated with culture medium (DMEM) or the siRNA transfection reagents without addition of siRNA (Optimem + Lipofectamine), or with scrambled siRNA, or two different siRNAs against caspase-9 (siCASP9 2428 and siCASP9 2429) as a control before induction of ferroptosis using erastin or RSL3. Primary FACS plots are demonstrated.

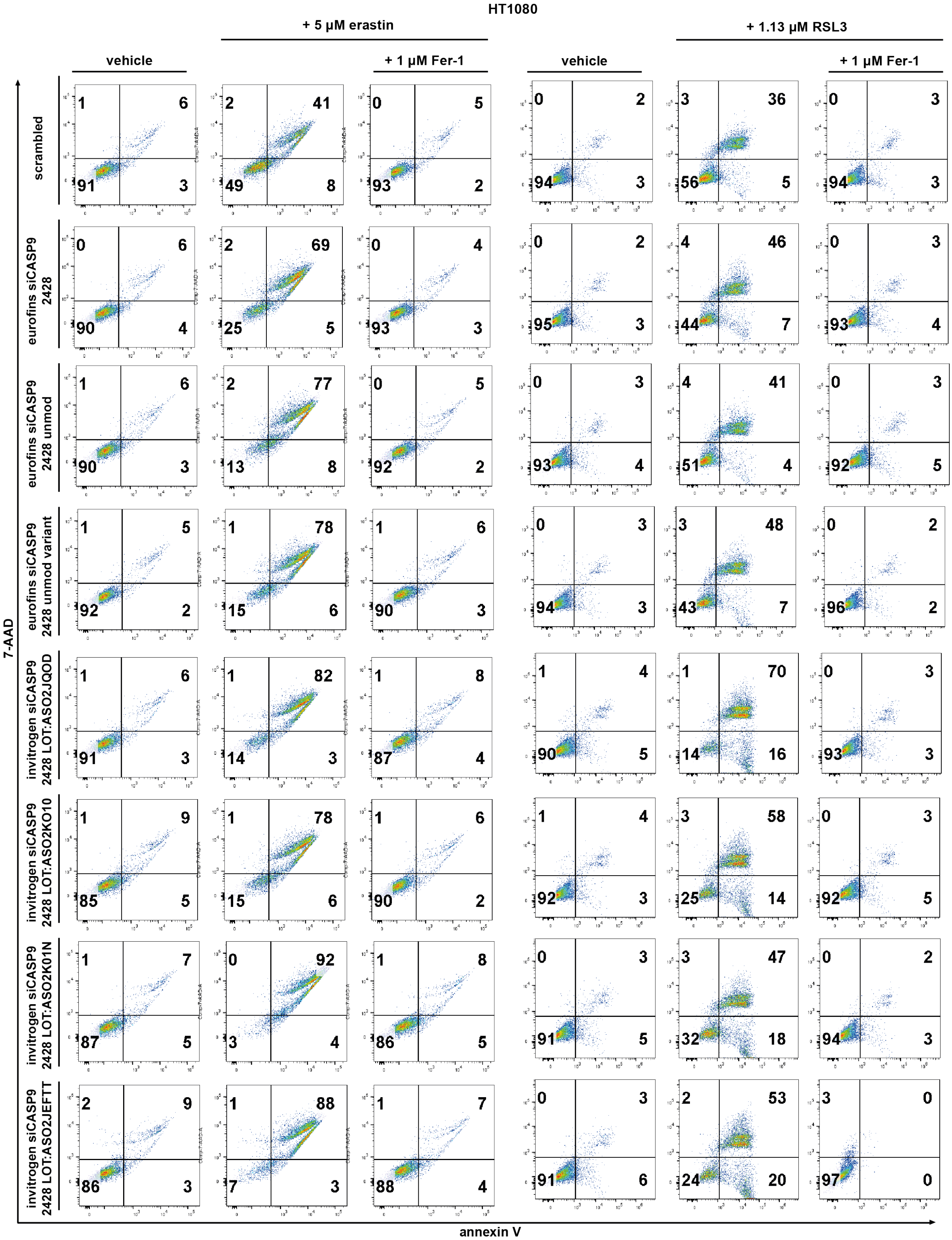

Figure S5. siRNA provider and Lot do not influence sensitization toward ferroptosis. HT1080 cells were treated with siRNAs from eurofins, that is siRNA against caspase-9 without locked nucleic acids (LNAs) (siCASP2428), one with the same sequence without LNAs (siCASP9 unmodified) or a variant where two nucleic acids were switched (siCASP9 unmodified variant), or with siRNAs against caspase-9 with LNAs (from Invitrogen) of different LOT numbers before induction of ferroptosis using erastin or RSL3. Primary FACS plots are demonstrated.

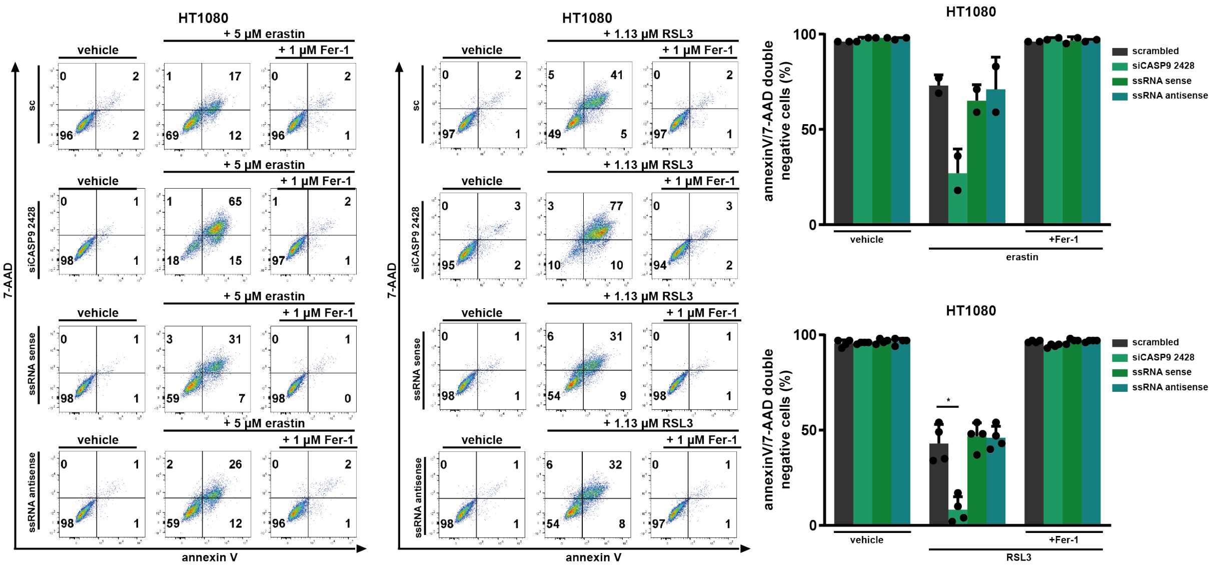

Figure S6. Other siRNAs against caspase-9 show different sensitization toward ferroptosis. HT1080 cells were treated with siRNA against caspase-9 or either only the ssRNA sense or antisense strand before induction of ferroptosis using erastin or RSL3. Primary FACS plots and respective quantifications of annexin V/7-AAD double negative cells are demonstrated. The graphs show means ± SD. Statistical analysis was performed using one way ANOVA. *p ≤ 0.05, ns: not significant.

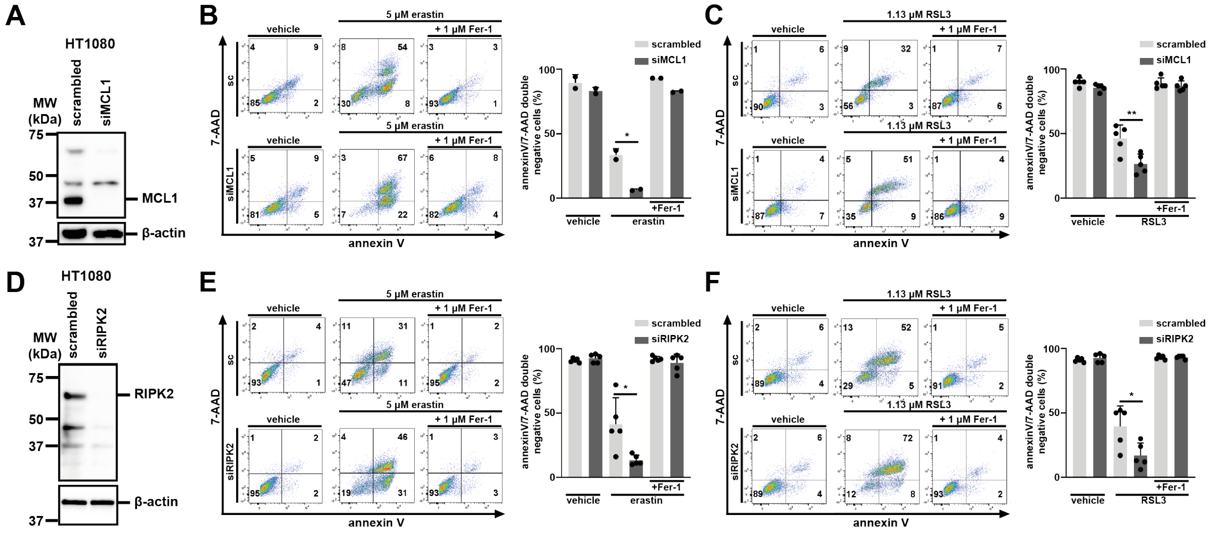

Figure S7. Transfection of commonly used siRNAs is associated with sensitization to ferroptosis. A) Western blot analysis to determine knockdown efficacy of MCL1. **B)** MCL1 was knocked down in HT1080 cells before treating them with erastin. 7-AAD and annexin V were read out by FACS. Primary FACS plots and respective quantifications of annexin V/7-AAD double negative cells are demonstrated. **C)** MCL1 was knocked down in HT1080 cells before treating them with RSL3. 7-AAD and annexin V were read out by FACS. Primary FACS plots and respective quantifications of annexin V/7-AAD double negative cells are demonstrated. **D)** Western blot analysis to determine knockdown efficacy of RIPK2. **E)** RIPK2 was knocked down in HT1080 cells before treating them with erastin. 7-AAD and annexin V were read out by FACS. Primary FACS plots and respective quantifications of annexin V/7-AAD double negative cells are demonstrated. **F)** RIPK2 was knocked down in HT1080 cells before treating them with RSL3. 7-AAD and annexin V were read out by FACS. Primary FACS plots and respective quantifications of annexin V/7-AAD double negative cells are demonstrated. Primary FACS plots and respective quantifications of annexin V/7-AAD double negative cells are demonstrated. The graphs show means ± SD. Statistical analysis was performed using Student's t-test. *$p \leq 0.05$, **$p \leq 0.01$, ns: not significant.

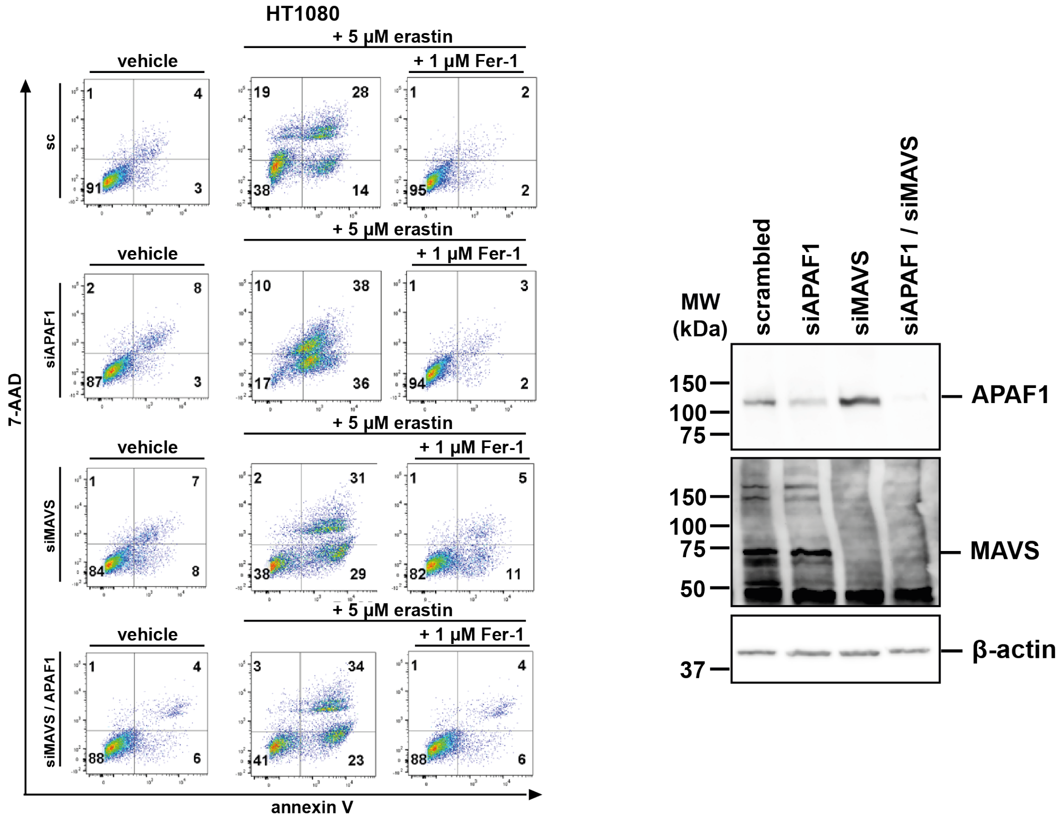

Figure S8. MAVS knockdown reverses the sensitizing effect to ferroptosis mediated by APAF1 siRNA. MAVS and APAF1 were knocked down in HT1080 cells before treating them with erastin. 7-AAD and annexin V were read out by FACS. Primary FACS plots as well as Western blot analysis to determine knockdown efficacy of MAVS and APAF1 are demonstrated.

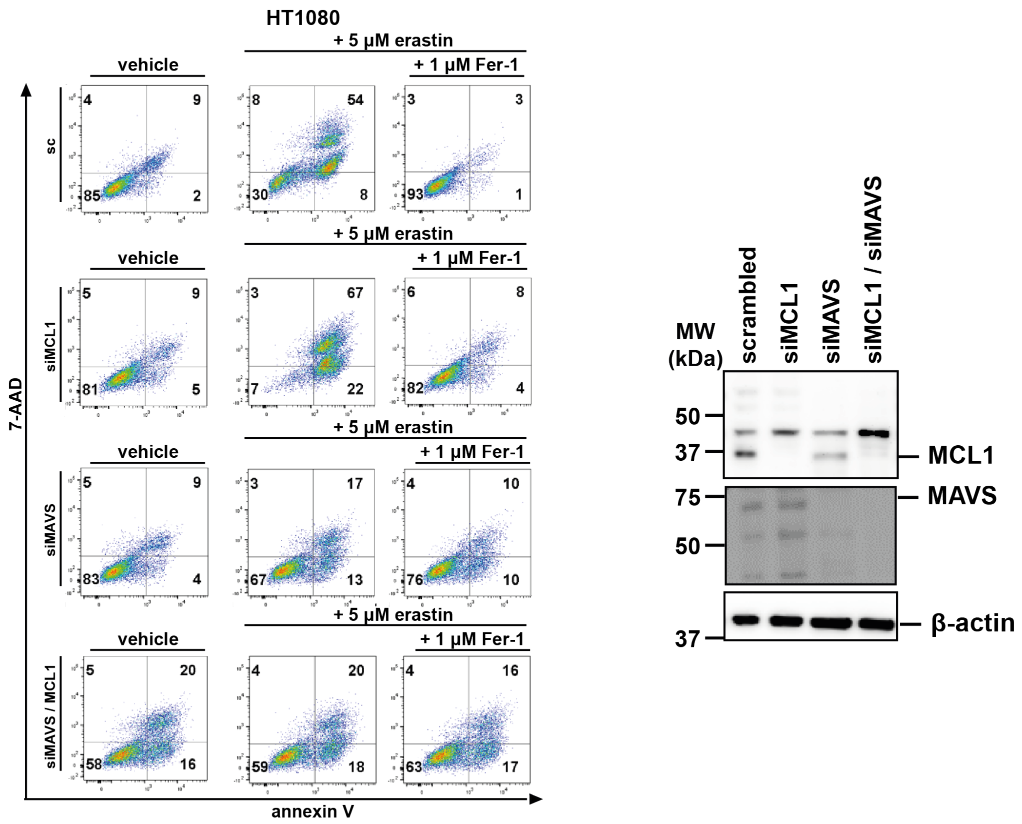

Figure S9. MAVS knockdown reverses the sensitizing effect to ferroptosis mediated by MCL1 siRNA. MAVS and MCL1 were knocked down in HT1080 cells before treating them with erastin. 7-AAD and annexin V were read out by FACS. Primary FACS plots as well as Western blot analysis to determine knockdown efficacy of MAVS and MCL1 are demonstrated.

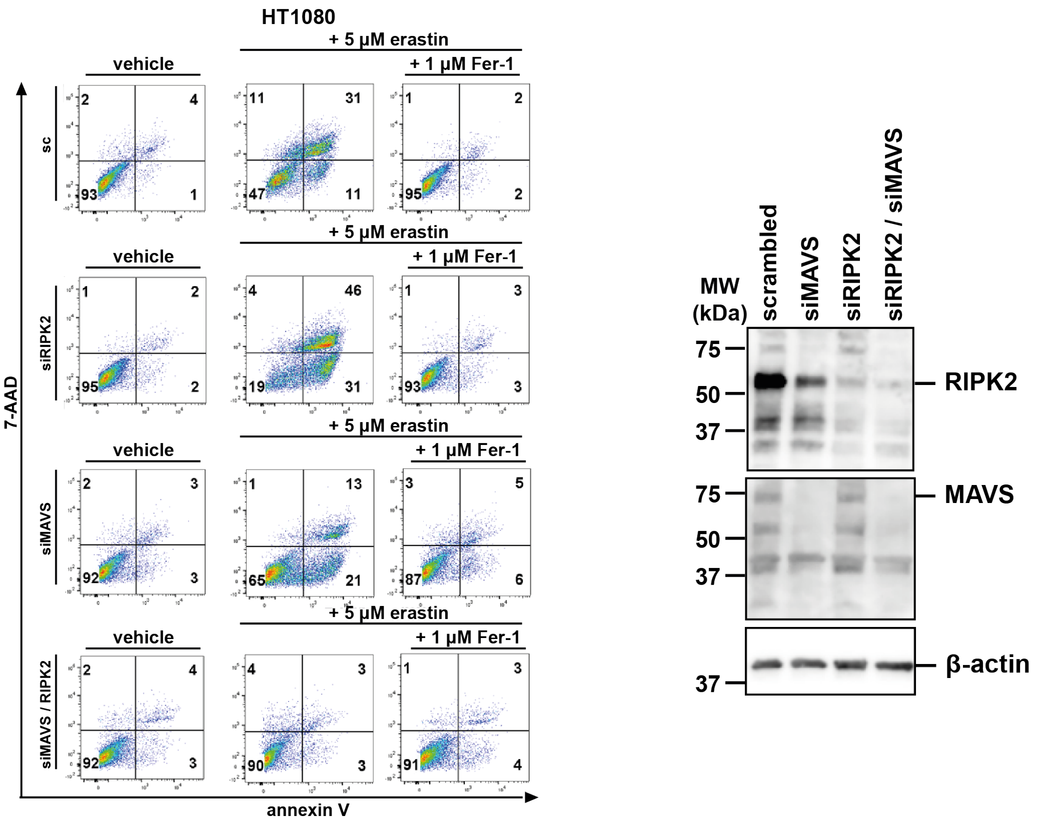

Figure S10. MAVS knockdown reverses the sensitizing effect to ferroptosis mediated by RIPK2 siRNA. MAVS and RIPK2 were knocked down in HT1080 cells before treating them with erastin. 7-AAD and annexin V were read out by FACS. Primary FACS plots as well as Western blot analysis to determine knockdown efficacy of MAVS and RIPK2 are demonstrated.

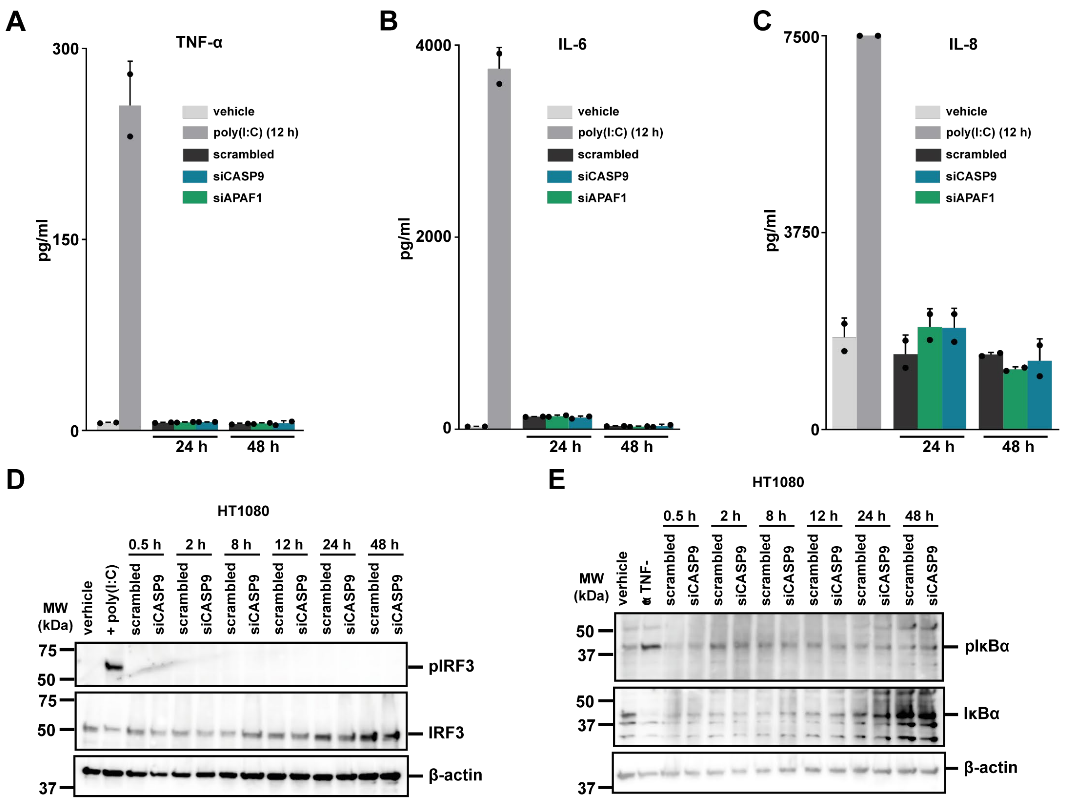

Figure S11: siRNAs fail to activate canonical proinflammatory responses downstream of MAVS. A) Caspase-9 or APAF-1 was knocked down in HT1080 cells before analyzing TNFα levels in the supernatant at indicated time points. Poly (I:C) was used as positive control. **B)** Caspase-9 or APAF-1 was knocked down in HT1080 cells before analyzing IL-6 levels in the supernatant at indicated time points. Poly (I:C) was used as positive control. **C)** Caspase-9 or APAF-1 was knocked down in HT1080 cells before analyzing IL-8 levels in the supernatant at indicated time points. Poly (I:C) was used as positive control. **D)** Caspase-9 was knocked down in HT1080 cells before analyzing pIRF3 protein levels at indicated time points. Poly (I:C) was used as positive control. **E)** Caspase-9 was knocked down in HT1080 cells before analyzing pIκBα protein levels and IκBα degradation at indicated time points. TNF-α was used as positive control. IκBα: nuclear factor of kappa light polypeptide gene enhancer in B-cells inhibitor, alpha, IL-6/8: interleukin-6/8, IRF3: interferon regulatory factor 3, MAVS: mitochondrial antiviral signaling protein, TNF-α: Tumor necrosis factor alpha.

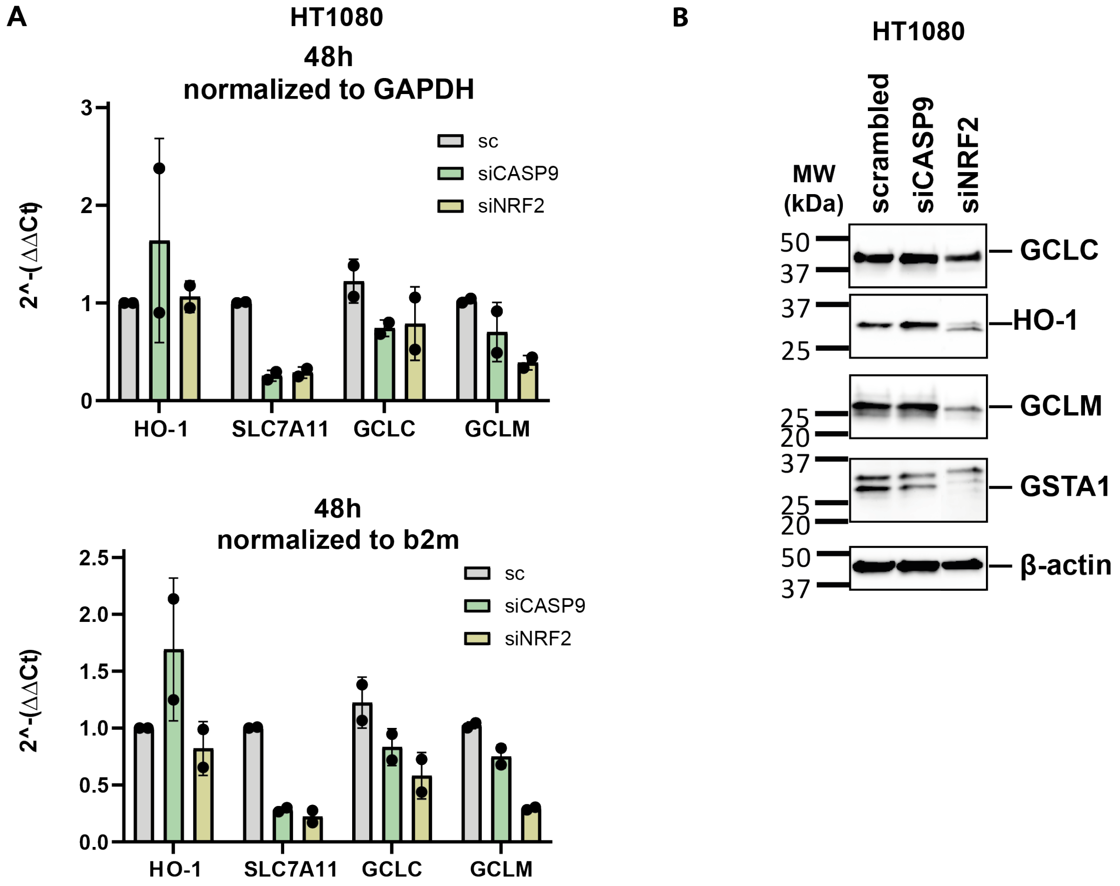

Figure S12: NRF2 target gene expression following siRNA treatment. A) HT1080 cells were treated with siRNA against caspase-9 or NRF2 before analyzing mRNA levels of the NRF2 target genes HO-1, SLC7A11, GCLC and GCLM at 48 h post transfection using qPCR. **B)** HT1080 cells were treated with siRNA against caspase-9 or NRF2 before analyzing protein levels of the NRF2 target genes HO-1, GSTA1, GCLC and GCLM at 48 h post transfection using Western blot analysis. HO-1: Heme oxygenase 1, SLC7A11: solute carrier family 7 member 11, GCLC: glutamate-cysteine ligase catalytic subunit, GCLM: glutamate-cysteine ligase modifier subunit, GSTA1: Glutathione S-transferase A1.
